# Combining unsupervised, supervised and rule-based learning: the case of detecting patient allergies in electronic health records

**DOI:** 10.1186/s12911-023-02271-8

**Published:** 2023-09-18

**Authors:** Geir Thore Berge, Ole-Christoffer Granmo, Tor Oddbjørn Tveit, Anna Linda Ruthjersen, Jivitesh Sharma

**Affiliations:** 1 Department of Information Systems, University of Agder, Kristiansand, Norway; 2 Department of Technology and eHealth, Sørlandet Hospital Trust, Kristiansand, Norway; 3 Department of ICT, University of Agder, Grimstad, Norway; 4 Department of Anesthesia and Intensive Care, Sørlandet Hospital Trust, Kristiansand, Norway

**Keywords:** Natural language processing, Electronic health records, Machine learning, Supervised, Unsupervised, Rule-based, Classification, Clinical decisions support systems

## Abstract

**Background:**

Data mining of electronic health records (EHRs) has a huge potential for improving clinical decision support and to help healthcare deliver precision medicine. Unfortunately, the rule-based and machine learning-based approaches used for natural language processing (NLP) in healthcare today all struggle with various shortcomings related to performance, efficiency, or transparency.

**Methods:**

In this paper, we address these issues by presenting a novel method for NLP that implements unsupervised learning of word embeddings, semi-supervised learning for simplified and accelerated clinical vocabulary and concept building, and deterministic rules for fine-grained control of information extraction. The clinical language is automatically learnt, and vocabulary, concepts, and rules supporting a variety of NLP downstream tasks can further be built with only minimal manual feature engineering and tagging required from clinical experts. Together, these steps create an open processing pipeline that gradually refines the data in a transparent way, which greatly improves the interpretable nature of our method. Data transformations are thus made transparent and predictions interpretable, which is imperative for healthcare. The combined method also has other advantages, like potentially being language independent, demanding few domain resources for maintenance, and able to cover misspellings, abbreviations, and acronyms. To test and evaluate the combined method, we have developed a clinical decision support system (CDSS) named Information System for Clinical Concept Searching (ICCS) that implements the method for clinical concept tagging, extraction, and classification.

**Results:**

In empirical studies the method shows high performance (recall 92.6%, precision 88.8%, F-measure 90.7%), and has demonstrated its value to clinical practice. Here we employ a real-life EHR-derived dataset to evaluate the method’s performance on the task of classification (i.e., detecting patient allergies) against a range of common supervised learning algorithms. The combined method achieves state-of-the-art performance compared to the alternative methods we evaluate. We also perform a qualitative analysis of common word embedding methods on the task of word similarity to examine their potential for supporting automatic feature engineering for clinical NLP tasks.

**Conclusions:**

Based on the promising results, we suggest more research should be aimed at exploiting the inherent synergies between unsupervised, supervised, and rule-based paradigms for clinical NLP.

**Supplementary Information:**

The online version contains supplementary material available at 10.1186/s12911-023-02271-8.

## Background

Natural Language Processing (NLP) driven clinical systems (e.g., decision support systems) have been heralded as an important brick for healthcare to deliver on the future expectation of precision medicine. Precision medicine is medicine tailor-made to each particular patient and their needs. A patient’s narrative may typically amount to hundreds or thousands of documents [1]. NLP has the capacity to search through unstructured text for clinical concept relevant information, tag and extract significant data, condense and synthesize such data into new information, and even aggregate it to new knowledge for advanced decision support [2].

Rule-based systems for NLP capable of extracting structured clinical information from the narrative were introduced into healthcare during the 1970s, and have been used successfully [3, 4]. Some well-known examples of rule-based systems used for clinical information tagging and/or extraction are MERKI, MedLEE (based), SymText, NegEx, MedEx, MedXN, MetaMap, KnowledgeMap, HITEx and ContextD [5–12]. However, while rule-based systems have a proven accuracy record, such systems may suffer performance-wise if words that appear in the narrative text are not accounted for in lexical resources [13, 14]. The medical language displays a range of different styles and grammatical structures, and it is opulent with abbreviations, compound words, lexical variants, and misspellings [15]. Thus, such systems are dependent on human clinical expertise for quality assurance of deterministic rules and the development and maintenance of specialized medical dictionaries used for clinical information extraction [16].

Machine learning-based systems for clinical NLP are a more recent phenomenon, with classification being a primary focus [17, 18]. Some well-known algorithms that have been implemented include Naïve Bayes, k-nearest neighbor (kNN), Conditional random field (CRF), Support Vector Machine (SVM), Logistic regression, Decision tree, Random forest, and artificial neural networks (ANN) [17–22]. However, despite there being a plethora of retrospective studies on supervised machine learning methods for NLP on anonymized patient data, only a limited number of prospective studies have been published, and relatively few such systems (e.g., Lancet, IBM Watson, and CTAKES being some notable exceptions) seems to have reached the maturity needed for practical implementation and impact [1, 2, 20, 23–31]. Indeed, supervised machine learning efforts in healthcare has been troubled with defending the cost of having clinical expertise annotate large amounts of narratives, which is necessary for even narrow-scope concept learning [32]. There is also the issue of privacy and confidentiality concerns, which hinders manual curation initiatives and excludes the sharing of annotated medical corpora [16]. Finally, supervised methods have also been criticized for being inaccurate, as they lack the fine-graininess of the deterministic rule-driven systems [33].

Provided enough data for extensive feature engineering, unsupervised machine learning requires no labeled data and may be used to learn meaningful representations of words from text [34–36]. Representation learning based on context-predicting methods received increased attention especially from 2013 when Mikolov et al. pioneered the Word2vec Continuous Bag-of-Words model (CBOW) and the Skip-Gram model for learning word representations [37–41]. More recent developments in this area are fastText, Global Vectors for Word Representation (GloVe), and StarSpace [37, 38, 42–45]. Large-scale unsupervised pre-training for global context embedding has lately been revolutionized by Transformer-based models such as bidirectional encoder representations from transformers (BERT) [46] and Generative Pre-training (GPT) [47].

These large neural networks consume huge amounts of unlabeled text corpora and learn nonlinear embeddings underlying the structure of the text. However, due to the scarcity of EHR data courtesy of privacy issues, limited availability of computational power and need for interpretability, using these large models is infeasible for critical applications in healthcare [48, 49]. Pre-trained word embeddings have been successfully used as extra word features in existing supervised NLP systems [50]. It has also been shown that training cluster-based word representations on domain corpora can improve classification performance versus, e.g., newswire-domain corpora [51–53]. However, there is still little research on employing real-life longitudinal EHR data to learn word representation for clinical NLP purposes [26, 53–59]. Although it has been suggested that state-of-the-art unsupervised semantic models may be utilized to automatically produce lexical resources, they do not constitute perfect representation-learning or similarity estimation engines [16, 60–63]. Consistent with the results of the classification experiments we perform, pure supervised and semi-supervised machine learning approaches (i.e., without also applying domain relevant rules) still struggle with achieving the high recall and precision rates that are required for patient diagnosis and treatment [18, 27, 64, 65]. Moreover, building high performing machine learning-based models requires powerful computing resources that are seldom available as part of contemporary healthcare institution IT infrastructure [66]. More complex algorithms, such as for instance those based on deep neural network principles, to varying degrees, can also be perceived as so-called “black boxes.” Once they are trained, it can be difficult to interpret why a particular response to a set of data inputs is given. This is certainly a disadvantage for healthcare, where decision making depends on transparency to achieve trust and understanding [67].

Evidently, none of the approaches described represent viable solutions for effectively and precisely searching the clinical narrative’s unique and rich vocabulary for information. As evidenced by recent clinical NLP challenges organized by the i2b2 Foundation, ensembles of complementary approaches generally improved performance, and the best-performing methods often used hybrid approaches that combined machine learning and rule-based methods [18, 27, 64, 65]. These systems could have multiple layers that leveraged the complementary strengths of each other, where the output of one method addressing portions of the NLP-related tasks could be a direct input to another adding both value and transparency. A challenge with the majority of these approaches, however, is their reliance on annotated datasets.

We present a novel hybrid-method for clinical NLP that leverages the combined synergies of unsupervised machine learning, semi-supervised machine learning, and deterministic rules. Unsupervised machine-learning is used in the pipeline architecture to automatically learn word representations from the clinical narrative and to build a knowledge base of the clinical language. Next, semi-supervised learning uses the word representations in the knowledge base to simplify and accelerate the building of specialized clinical vocabulary and concepts. In brief, this constitutes an interactive labeling process, where word clusters of related word representations are visualized in a two-dimensional Treemap structure that clinicians can manipulate dynamically to shape specialized clinical vocabularies. Built vocabularies can next be used and reused as building blocks to synthesize multiple clinical concepts (e.g., patient allergy) used for clinical information extraction. Because the underlying vocabulary is derived directly from the clinical narrative itself, the interactive process of building such clinical concepts represents an alternative accelerated and transparent approach to traditional manual feature engineering and tagging. Finally, a precision layer of deterministic rules is implemented for each concept to allow fine-grained control of information extraction from the narrative. The rules are domain comprehensible and post-process the output of the machine learning algorithms for achieving rule-based system level recall and precision at run-time. The integrated method takes advantage of each method’s strengths, while minimizing inherent weaknesses. The clinical language is automatically learnt, and vocabulary, concepts, and rules supporting a variety of NLP downstream tasks can be built with minimal manual feature engineering and tagging required from clinical experts. The method’s open processing pipeline architecture gradually refines unstructured (i.e., unlabeled) information into structured (i.e., labeled) data, which is different from a “black-box” type approach. Imperative for healthcare, data transformations are thus made transparent and predictions interpretable. The combined method also has other synergistic effects, like potentially being language independent, automatically updatable (meaning minimum manual maintenance is necessary for the algorithms and the built vocabularies and concepts), and able to cover misspellings, abbreviations, and acronyms.

To test and evaluate the method, we have developed a clinical decision support system (CDSS) named Information System for Clinical Concept Searching (ICCS) that implements the method for clinical concept tagging, extraction, and classification. The system’s performance has been empirically demonstrated in two previous papers. The system delivers high recall and precision rates (recall 92.6%, precision 88.8%, F-measure 90.7%), and the results of a recent field trial indicates high degrees of system acceptance among its clinical users [1, 68].

While the two earlier studies are important in that they give a preliminary outline of the system’s architecture, components, and user interface [1, 68], and also prove the system’s usability in clinical practice [68], this paper’s attention is first on describing the system’s underlying method. Second, complementing limited earlier research and validating the method proposal, we perform experiments with various unsupervised methods on the task of word similarity to evaluate their capability for feature engineering; i.e., whether the models produced can serve as the base for further semi-supervised building of specialized clinical vocabularies. Third, the performance of the proposed combined method is evaluated on the task of classification (i.e., detecting patient allergies) against a range of common supervised learning algorithms. Finally, we analyze and discuss the results of the evaluation tasks, and we interpret the results in the context of clinical NLP.

## Methods

The combined method we propose for clinical NLP was motivated by the possibilities and limitations outlined in the Background section. We experimented with orchestrating different methods for accurate and effective clinical concept tagging and extraction. Being aware of the inherent challenges that go with manual annotation of medical datasets, we aimed to devise a method independent of such means by leveraging on machine learning’s potential for automatic feature engineering.

**Fig. 1:**
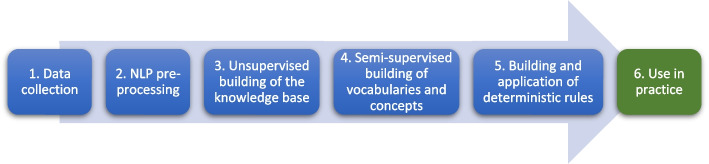
The pipeline architecture for the combined method as a series of sequential steps

The pipeline architecture for the combined method has been designed for deployment in a hospital production environment (i.e., ICCS) [69]. The architecture includes a series of sequential steps, as depicted in Fig. 1. Except for the first subsection that provides a short introduction of the architecture, modules, and functionality of the CDSS we have developed, the rest of this section is organized in accordance with the pipeline architecture and explains each step successively.

### System architecture, modules, and functionality

**Fig. 2:**
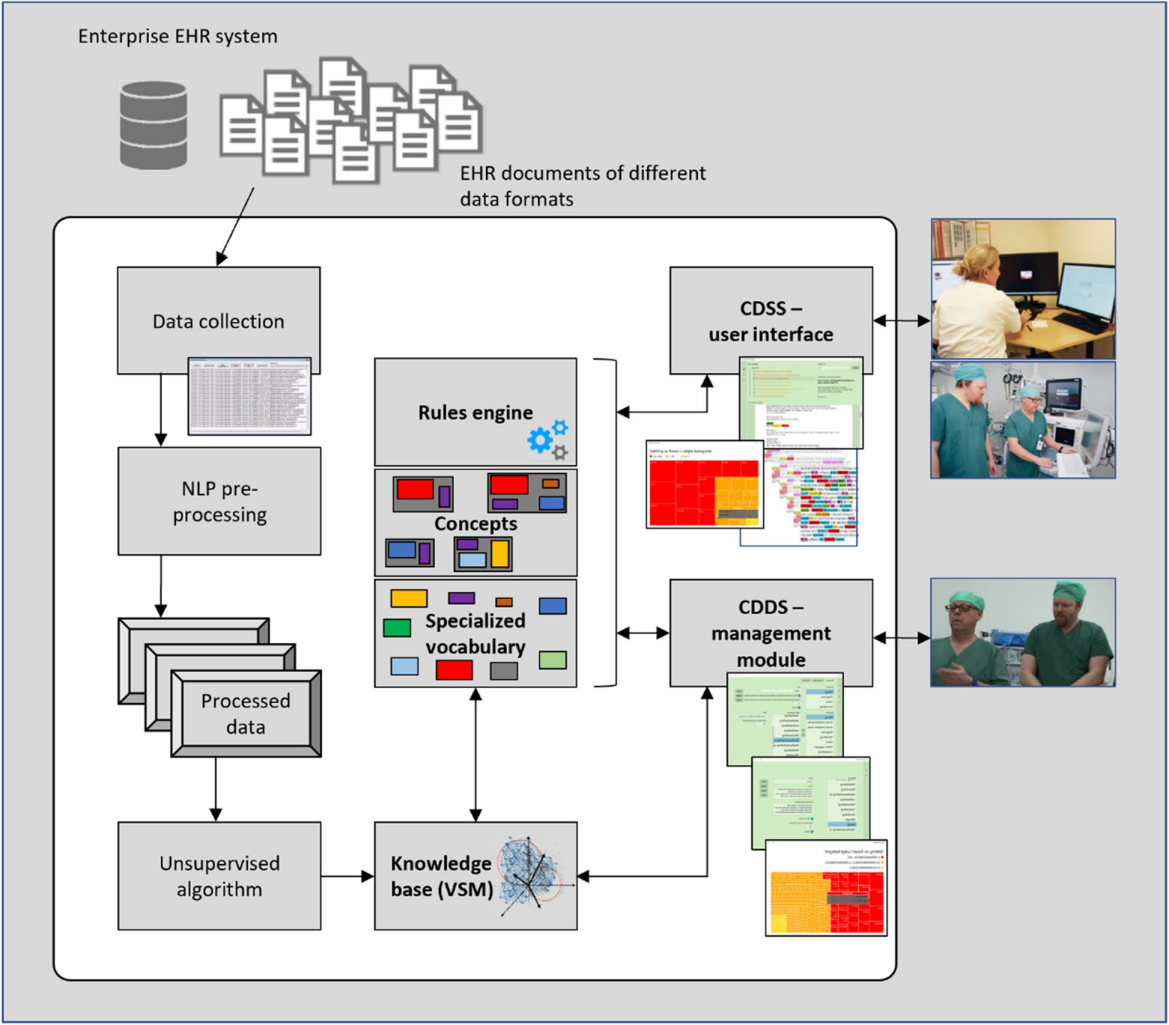
A simplified overview of the system achitecture for building and using specialized clinical vocabulary, concepts, and rules. The management module is related to step 4 and 5 of the pipeline architecture, while the user interface is related to step 6 (step 1–3 are shown on the left)

Figure 2 shows a simplified overview of the system architecture implemented for building and using specialized clinical vocabulary, concepts, and rules.

The main functionality of ICCS is its capability for clinical concept building, searching, and classification by processing unstructured information (i.e., the narrative) and structured data (i.e., laboratory results and critical information) in EHRs. At run-time (step 6 in Fig. 2), the system typically provides the user (e.g., a doctor or nurse in the hospital) with lists of documents, laboratory results, and critical information that are patient specific and relevant to a particular use-case (e.g., drug allergies, infectious diseases, or patient diagnoses) defined by a set of seed words (the term “word” may hereafter also be used in the meaning of “phrase”) and rules. The system is further able to identify and extract clinical concept relevant information with context to make it meaningful, and map that information into a structured representation. For example, using the clinical concept of “drug allergies,” the system can identify “Penicillin”, and provide context such as whether a patient is allergic to the drug. While the system’s main processes and modules are depicted in Fig. 2, we refer to [1, 68] for further presentations of system design, development, and functionality.

### Data collection

The pipeline architecture with all of its steps, including the data collection component (step 1 in Fig. 1) briefly described here, must support volume, variety, and velocity, which also are the three defining properties of big data [70, 71]. Data collection needs to be able to keep up with the real-time production of data in the clinical environment (i.e., velocity), which typically may amount to thousands or tens of thousands of documents each day in medium- or large-sized Norwegian hospitals. Methods for calculating deltas on large amounts of data must be designed to support the continuous export of fresh data with as little latency as possible. The production environment databases or data warehouses must also allow real-time querying of both structured data (demographics, vital signs, etc.) and unstructured information such as clinical notes.

Data collection also needs to be able to accommodate the large volumes of historical data that is typically stored in such hospitals’ contemporary EHR systems, where data volumes commonly amount to tens or hundreds of terabytes. For example, in a previous study, we evaluated our method in a middle-sized Norwegian hospital [68] that has a daily production of about 11 000 new documents and stores about 67 million documents, or 23 terabytes of data, only in its enterprise EHR system.

With reference to variety, contemporary EHR systems may contain a range of different data or document formats. Most EHR systems also scan documents and store these in different image formats. Because NLP techniques and ML algorithms typically only support processing of plain text, the content of documents or images must be extracted and transformed into raw text before further processing. Legacy document formats, or proprietary vendor-specific formats, may further require reverse engineering of composition and content and the development of custom-built solutions for extraction and transformation of text. A subset of the most common document types and database datatypes we encountered when we exported documents from the hospital’s enterprise EHR system is listed in Table 1.

**Table 1:** A subset of the document types and database datatypes stored in the hospital’s enterprise EHR system

Data formats	Oracle datatypes	Document type examples
RTF	CLOB	Discharge summaries, surgery notes, journal notes
PDF	CLOB	Laboratory results, anesthesia and intensive care journals
Formflow	CLOB	Specialized forms, surgical nurse reports, medical certificates
TIFF	BLOB	Scanned documents, clinical curve data
XML	XML	Physician referrals, CAVE/drug reactions
HTML	VARCHAR	Physician referrals
Proprietary/legacy data formats	VARCHAR	Specialized forms, pre-operative assessments and planning forms
Text	VARCHAR	Older documents from converted legacy EHRs

The data collection step, with all of its sub-processes, must be streamlined, integrated, and automated to prepare data for further NLP preprocessing and the later steps. Finally, the processes must be able to execute without causing disturbances to the EHR systems so as not to result in catastrophic system errors or delays (i.e., potentially affecting patient treatment negatively).

In the previous evaluation study [68], patients’ EHR data and documents (transformed into raw text format) were continuously sampled and imported into a document database for further pre-processing, text mining, and building of machine learning models. As no off-the-shelf solution capable of supporting the necessary extraction and transformation of data from the EHR system was available, we developed several customized C# and Python-based software solutions to automate and integrate these steps for near real-time execution.

### NLP pre-processing

Unlike structured data, where machine learning algorithms can operate directly on the underlying data, text in EHR documents requires preprocessing before machine learning algorithms can be successfully applied. The NLP pre-processing stage (step 2 in Fig. 1) uses several methods including lowering case, removal of punctuations, sentence boundary detection and splitting, and tokenization. Stop words are not removed, because these are important for determining word co-occurrence central to word embedding. We consider a token to be any sequence of symbols, separated by white space. Additionally, N-gram (unigram, bigram, trigram, and quadrigram) models necessary for machine learning feature generation are built by performing chunking of tokens [72]. We designed the NLP pre-processing through an iterative process where the medical domain knowledge of the language and vocabulary played a central role.

### Unsupervised building of the clinical language knowledge base

**Fig. 3:**
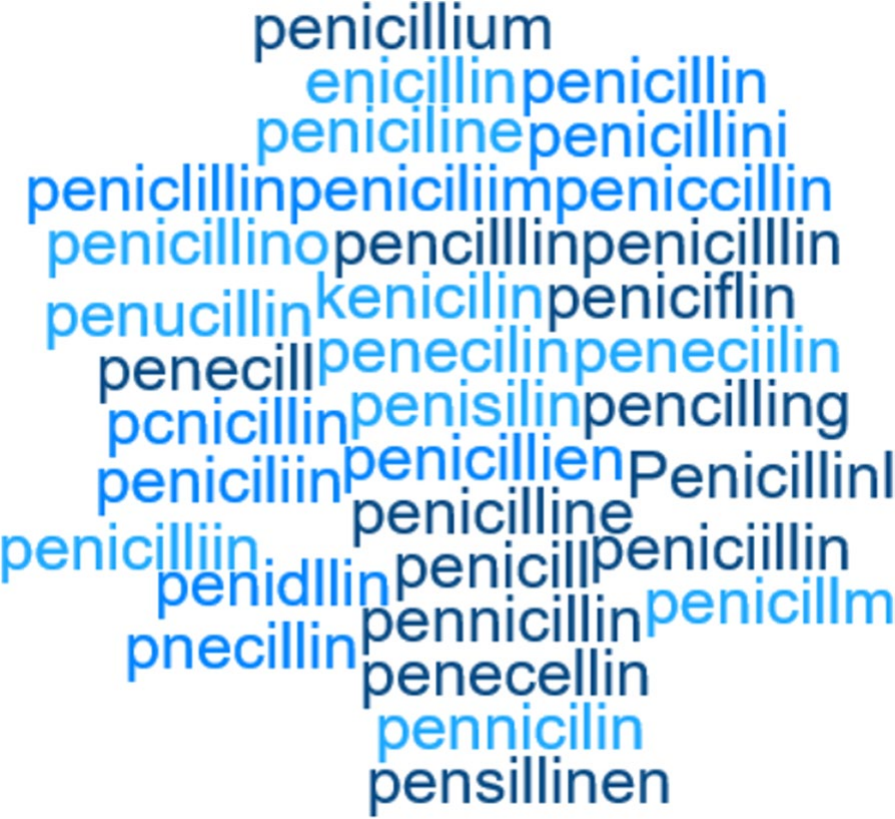
Word cloud depicting the drug name “Penicillin” and the 33 misspelled variants identified in the dataset

Even though great efforts have been made to standardize medical language (e.g., the use of international medical classification systems such as the Systematized Nomenclature of Medicine Clinical Terms [SNOMED-CT] and the International Classification of Disease [ICD]), there are still variations between healthcare providers depending on, e.g., medical domain specializations, culture, equipment and techniques, and employees’ professions and background, as well as misspellings and medical acronyms present in the narrative. To illustrate the amplitude of the misspellings challenge, Fig. 3 depicts the 33 misspelled variants of the drug name “Penicillin” identified by implementing a simple 4-edit distance limited algorithm on the 863 937 clinical notes included in the dataset we use to conduct experiments. Because we only use about 2% of the available hospital narratives to train on in our experiments, increasing the dataset size would likely result in the detection of even more misspelled variants. Missing out on only one of the variants could potentially be fatal for patient treatment depending on the use scenario of a downstream clinical NLP system. We observed similar misspelling rates for most of the key medical terms we examined. To facilitate high levels of precision and recall in downstream clinical NLP tasks, it is necessary to learn the unique features of the medical language in local context (i.e., a specific healthcare organization) [73]. Because manually labeling the narrative is not feasible, we devise the use of unsupervised learning to map local context medical language (step 3 in Fig. 1).

**Fig. 4:**
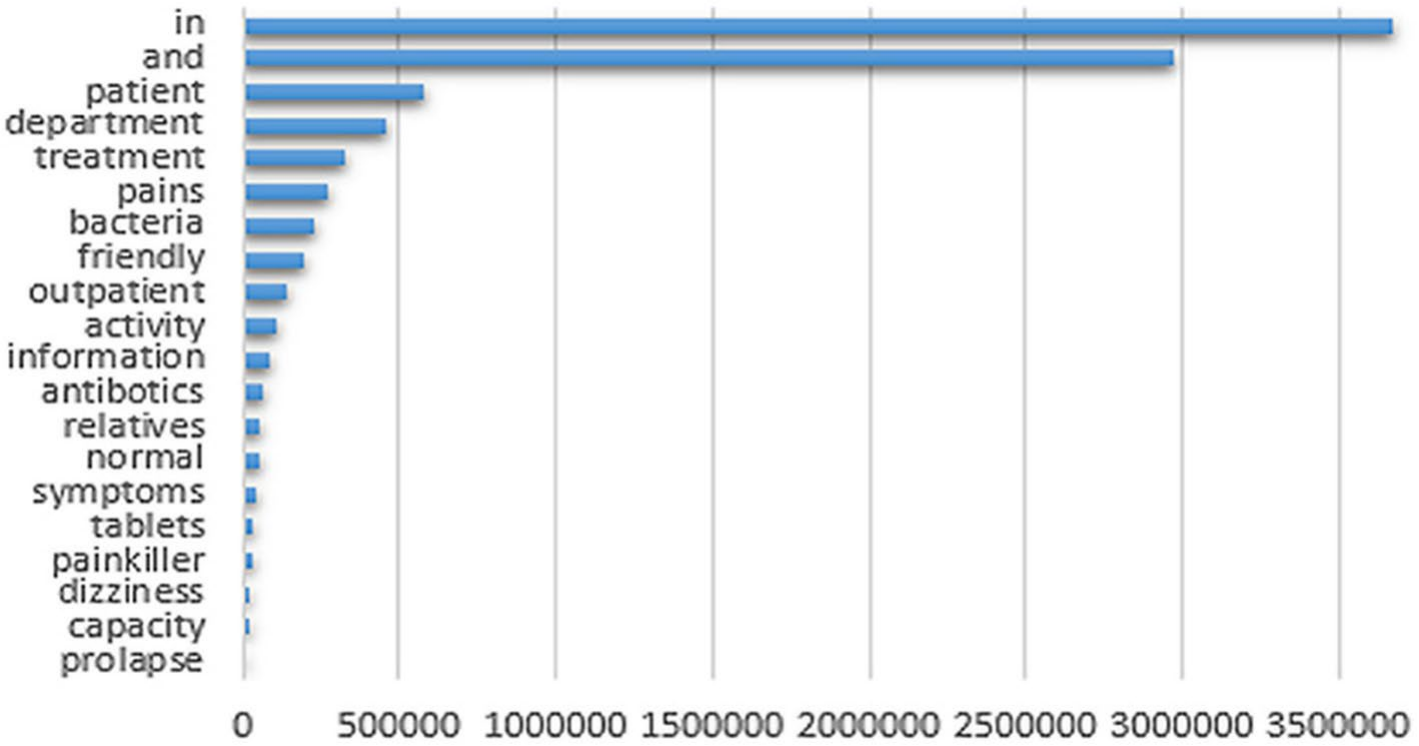
Zip's eponymous law [76]. Illustrated by a selection of word counts based on the analysis of the 863 937 clinical notes included in the dataset we use to conduct experiments, which yielded 863 937 unique unigram tokens and 1 803 428 common phrases in the knowledge base. Frequent words account for a large percentage of the text, but a large portion of words appear at a low frequency

Considering that texts are largely made up of rare words (see Fig. 4), the assumption of normal distribution may limit the ability to analyze and learn corpora in an unsupervised way [74–76]. However, the empirical experiments we conduct (see the Experiments section) reflect that the volumes of clinical data available in most healthcare organizations are sufficient for such methods to produce semantic vector space models (VSMs) that can effectively serve as knowledge bases to facilitate accelerated supervised building of specialized clinical vocabulary [77].

**Fig. 5:**
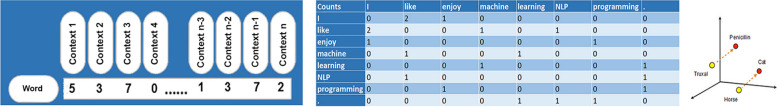
Transformation of words into vectors in the VSM. In the figure to the left are shown how words are represented as vectors according to contextual (document) frequency. In the table in the middle is shown an example of a word-context matrix (for the sentences “I like machine learning,” “I like NLP,” and “I enjoy programming”) where unique corpus relevant co-occurring words are counted on a global scale. Finally, to the right is shown an example of how closely related words vectors relate to each other in the continuous vector space

By using the explicitly encoded linguistic regularities and patterns stored in such word embedding vectors, the semantic meaning of words can to some extent be captured. As depicted in Fig. 5, the vector of each word contains components that capture contexts that characterize how it is used. As further illustrated to the right in Fig. 5, drugs such as “Truxal” and “Penicillin” are likely to share the same neighborhood of co-occurring words in clinical narratives (e.g., “he reacted to Penicillin”, “she reacted to Truxal”).

**Fig. 6:**
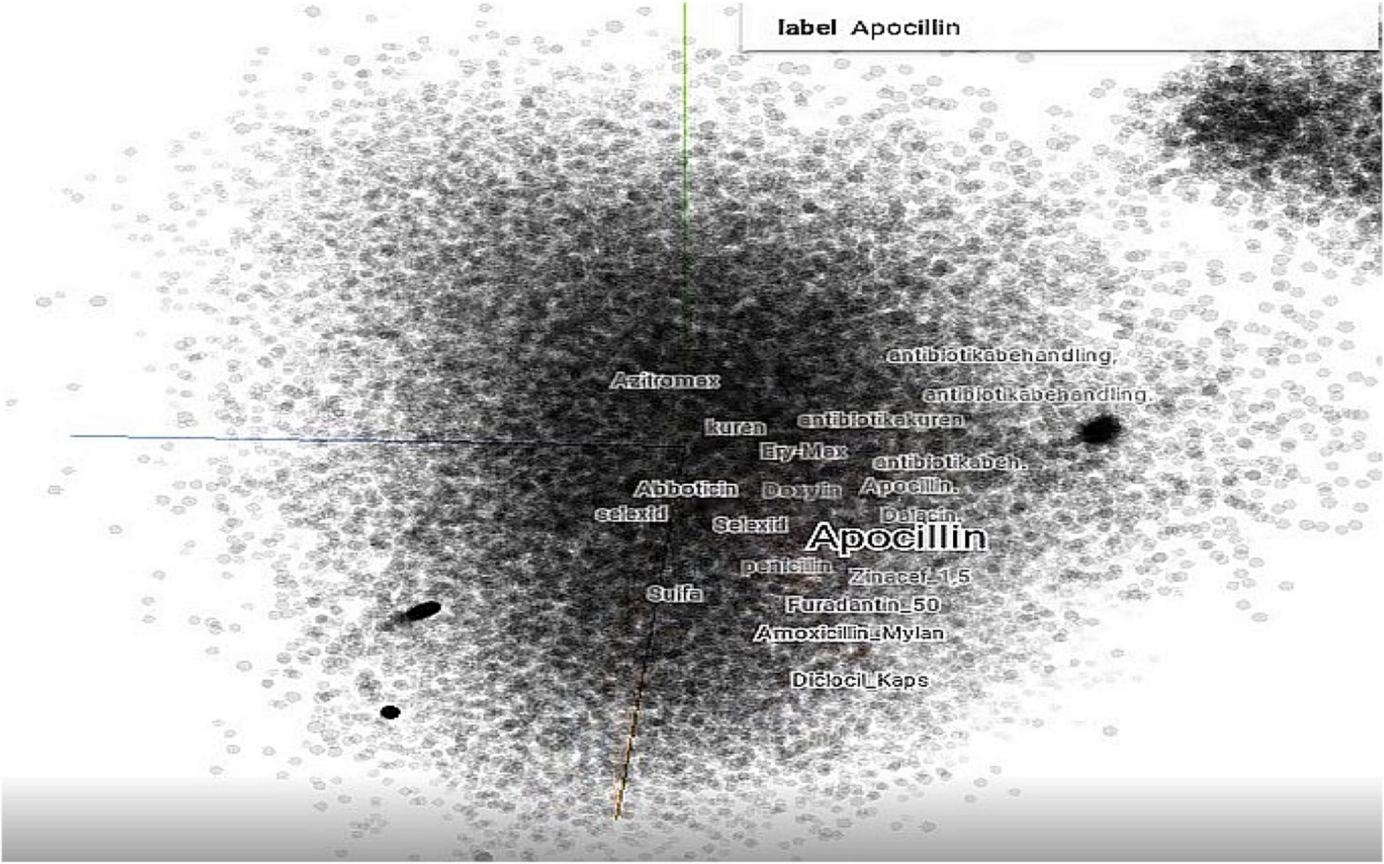
Exploring one of the clinical language models as a 3D VSM using a Tensorflow implementation. Using “Apocillin” as the seed word, the nearest neighboring words are shown. The model may be twisted and zoomed in and out to explore word relations

Figure 6 shows a visualization of one of the clinical language models trained on data from authentic EHRs from Sørlandet Hospital Trust. We observe that semantically similar words are typically mapped to nearby points, reflected by their relatively small angle or short distance between each other (see also Table 3 in the Experiments section).

The specific word embedding method (see Algorithm 1, activity A) that we implement in the combined method (i.e., as part of the CDSS and the experiments here) was developed by one of the authors and uses global matrix factorization and local context window method [34, 37, 43]. While grammar and word order are disregarded, features such as frequency of words, word location, and word co-occurrence related to the usage of words are extracted and encoded in word-context matrixes. Our method implements probabilistic clustering technique based on Bayesian reasoning for prediction. This allows the confidence in the prediction to be quantified as a predictive distribution instead of relying on a single number (i.e., the “dot product”) [78], which helps with vagueness (an entity may be equally applicable to multiple points in the VSM) and gradedness (an entity may be more applicable to some points in the VSM than to others). The present paper has a system and applied perspective, focusing on how the underlying methods combine and interact to solve a challenging real-world problem. We refer to Berge et al. [1] for some more details on the method. Our encouraging results with producing word embeddings (see Table 3 in the Experiments section) demonstrate that any of the included word embedding methods may potentially be used to build a VSM of the medical language in patient EHRs.

**Figure:**
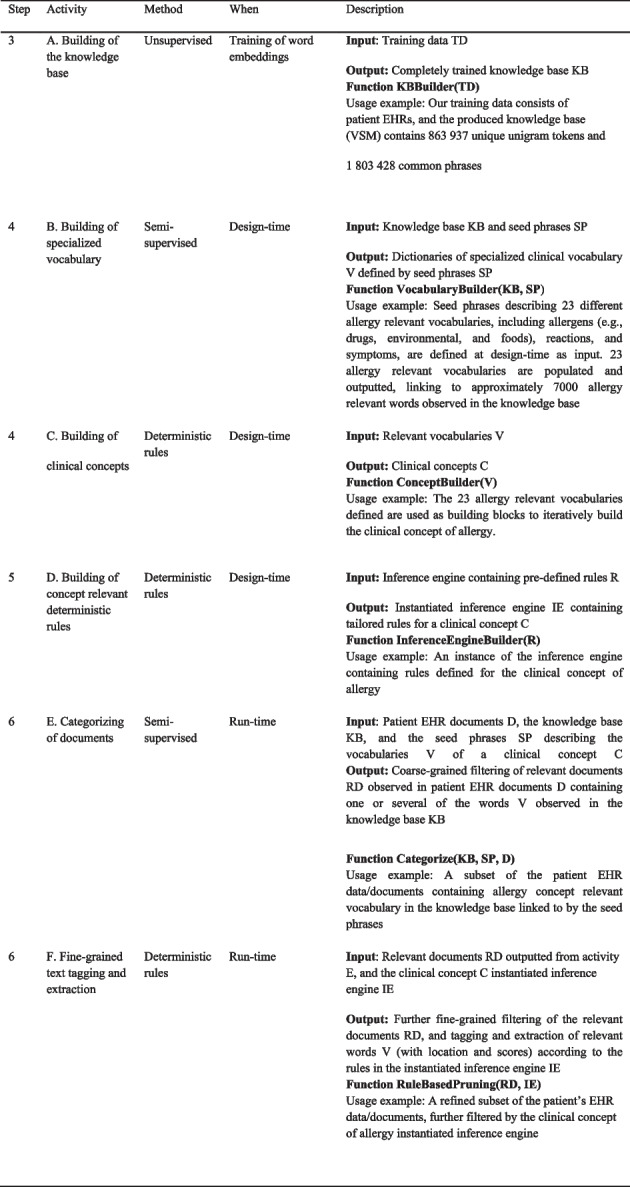
**Algorithm 1.** Details of activities included in step 3–6 in Fig. 1 described as algorithms. The provided usage examples demonstrate how the activities in the steps have been implemented in ICCS to detect, e.g., relevant allergy information in the clinical narrative.

### Semi-supervised building of vocabulary and clinical concepts by querying the knowledge base

Lexical repositories have proven to be very useful resources for NLP [79]. However, despite the recent progress with producing unsupervised semantic models, they are not able to automatically generate perfect lexical resources [16, 60–63]. While it is an easy task for humans to measure semantic distance (i.e., closeness in meaning) of two concepts, it represents a challenge with such models in particular because of word sense ambiguity [62]. A large portion of words correspond to several unrelated meanings (i.e., are homonyms) or multiple related senses (i.e., are polysemes), which is problematic with most word embeddings that typically only represent each word with one vector. For example, the word “sign” may both convey an objective finding of a disease state (e.g., fever or rash), a plus or minus indication in math (e.g., laboratory tests), and meaning like in the sentence “she took it as a sign” (i.e., in the psychiatric narrative).

To alleviate the shortcomings intrinsic to pure unsupervised approaches, the combined method we propose makes use of an optimized word embedding model to create dictionaries of specialized clinical vocabulary semi-automatically (step 4 in Fig. 1). Using a vector distance measure such as cosine, relevant categories of positive seed words (e.g., drug names, diagnoses, diagnostic procedures, medical conditions, contagious diseases, implants, laboratory tests, and radiology exams) are used as manual input to query a word embedding model for candidate similar words. The similarity of two words (e.g., seed word w1 and word w2) in a VSM can be computed as the inner product between their vectors,

1 $$Sim\left(w1, w2\right)=\mathrm{cos}\left(\theta \right)=\frac{w1 \bullet w2}{\Vert w1\Vert \Vert w2\Vert }=\frac{{\sum }_{i}{w1}_{i}{w2}_{i}}{\sqrt{{\sum }_{i}{w1}_{i}^{2}}\sqrt{{\sum }_{i}{w2}_{i}^{2}}}$$

where the simple range of cosine coefficients from -1 to 1 may be thought of as correlation coefficients that can be used for two-dimensional projection of word embedding vectors. A value of 0 means words are orthogonal (90 degrees) and completely unrelated, a value of 1 means they share the same orientation and is perfectly related, while a value of -1 represents a perfect opposite relationship (180 degrees).

**Fig. 7:**
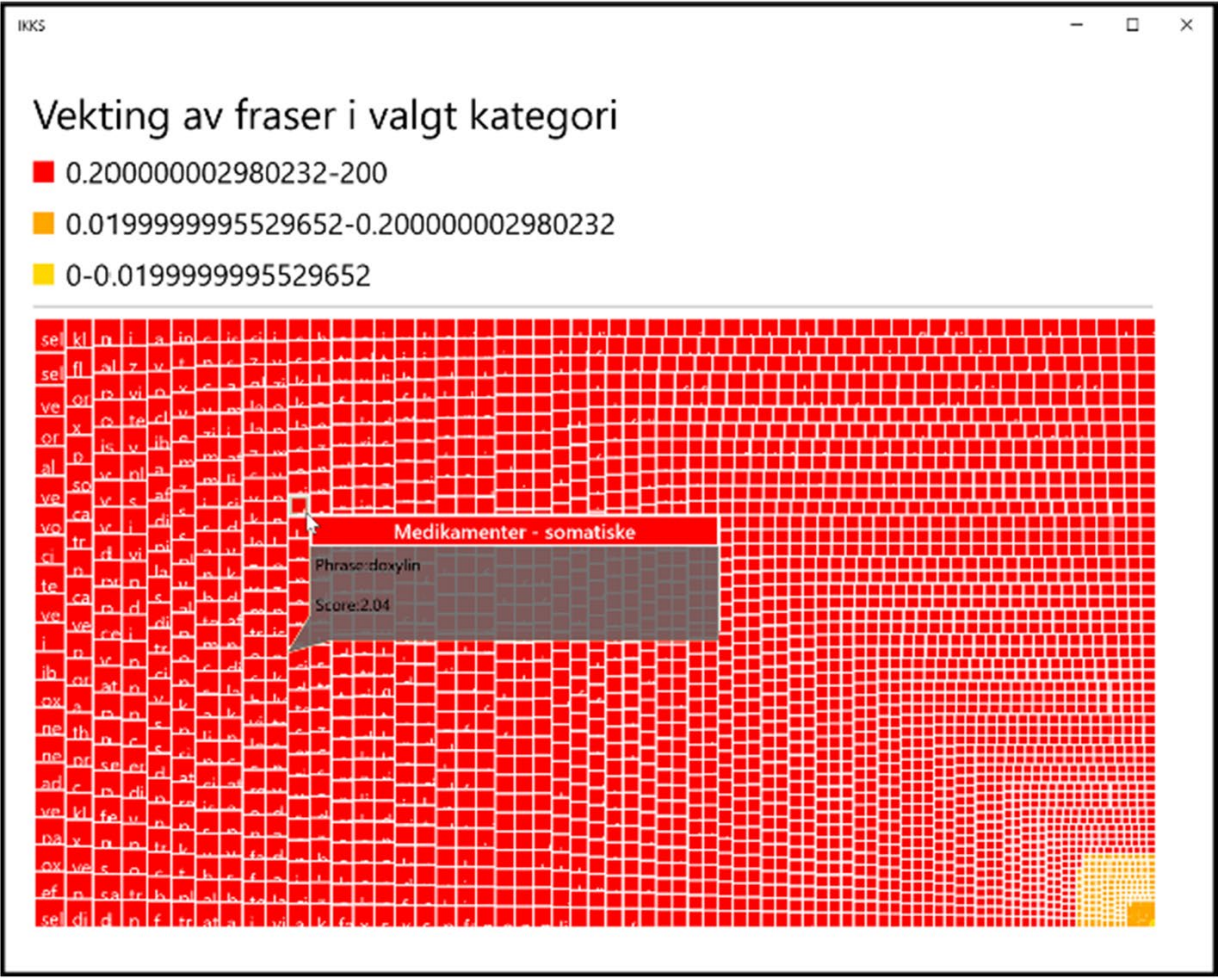
A Treemap structure of 1853 drug relevant phrases returned by the system (text in Norwegian)

More specifically, the system we have developed features a management module (see Fig. 2) that simplifies and accelerates the building of both vocabularies and concepts by human supervision (e.g., a medical domain expert). The system makes use of nested rectangles with different colors, sizes, and scores in a Treemap structure to intuitively visualize word similarity (see Fig. 7). That is, both the colors and area sizes of the candidate words reflect vector score or relatedness to positive seed words. This represents a further simplified abstraction of the cosine word similarity measure, more suitable for human manipulation than word vectors in a complex VSM model.

In brevity, when starting to build a new specialized vocabulary, relevant seed words (e.g., three drugs) are entered in the graphical user interface (GUI) as input (see Algorithm 1, activity B). The words are next translated into their embedding vectors. The knowledge base is queried, and the value of the input vectors are compared to stored vectors in the knowledge base to identify the corresponding nearest matches. Clustering is performed in real-time, to group sets of coherent vectors in the knowledge base. Having established all of the relevant clusters with their boundaries, the words included in the clusters are returned as candidate vocabulary relevant words (e.g., all of the 1853 drug names occurring in the dataset used for training word embeddings; see Fig. 7).

Further, similar to e.g. the programmatic implementation of the Word2vec Python Gensim library [37, 80], the returned list of candidate words can be semantically shaped (i.e., widened or shrinked) interactively by having the user introduce multiple positive and negative words until the required level of recall and precision is achieved (see Algorithm 2). Lists of candidate similar words can also be pruned for example by limiting the number of returned top n ranked words or by returning only supra-threshold (specified) cosine values. All the word embedding models we experiment with (see the Results and discussion section) can be manipulated in a similar manner.

**Figure:**
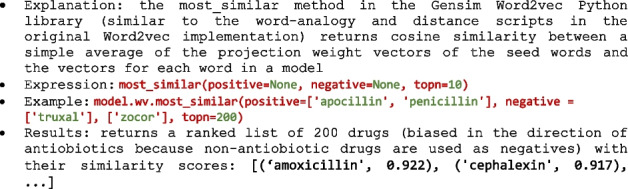
**Algorithm 2.** Querying the VSM to shape vocabularies.

The system also has the ability to let users explicitly add words in the case they are not present in the word embedding model, and to remove words if the described pruning methods are not successful (e.g., sometimes a specific negative seed word that is introduced may overlap with several adjacent word clusters, and the result is pruning of more similar words than desired).

The vocabulary building process is an iterative process that ends when all the vocabularies that define a clinical concept has been created. Once built, vocabularies can be reused as building blocks in multiple clinical concepts. The user builds a clinical concept by selecting the vocabularies that define it as building blocks (see activity C in Algorithm 1). For example, the concept of “allergy” contains 23 specialized vocabularies including allergens (e.g., drugs, environmental, and foods), reactions, and symptoms.

**Fig. 8:**
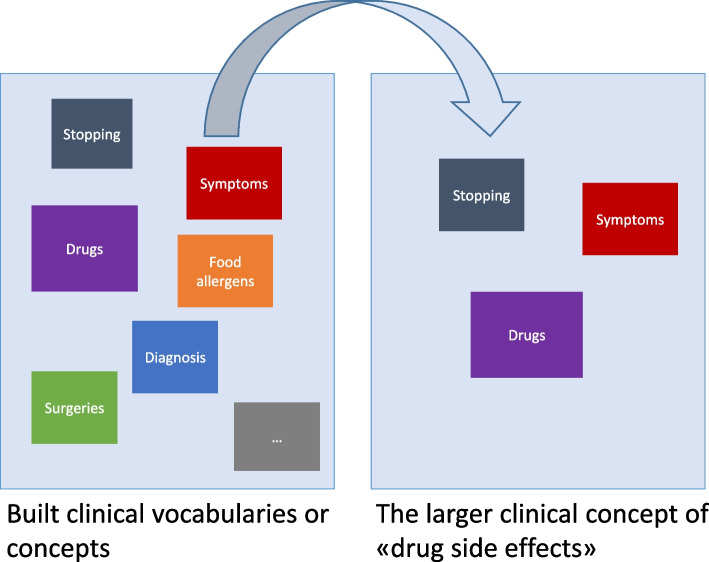
An illustration of the simplified and accelerated process of building clinical concepts in the developed CDSS, building higher-order hierarchical medical concepts, and showing three lower-order medical concepts being reused and pooled together to build a higher-order hybrid medical concept (corresponds to the “Concepts” object in Fig. 2)

Finally, the system contains functionality to pool several clinical concepts together into larger hybrid- or higher-order hierarchical clinical concepts (see Fig. 8). For example, the higher-order concept of “alert information” typically contains the five lower-order concepts allergy, infectious diseases, special disorders (e.g., haemophilia, angioedema, Addison's disease, porphyria), implants, and previous complications associated with anesthesia. Thus, creating new larger hybrid clinical concepts, containing several smaller refined clinical concepts with specific clinical vocabulary, becomes an accelerated process with the possibility of reusing already fabricated building blocks in various configurations. As further described in the next section, precisely searching for and classifying a built clinical concept at system run-time depends on the fulfillment of a set of rules also defined by the user at concept design-time.

### Using deterministic rules for precision-based search and classification results

A literature study was conducted to identify common deterministic rules used for clinical NLP purposes. Through an iterative process of applying domain knowledge and using semi-supervised built clinical concepts (step 4 in Fig. 1), we identified a final set of 35 deterministic rules that can be applied to augment the machine learning-based methods used for clinical concept searching and classification (step 5 in Fig. 1). These rules (see activity D and F in Algorithm 1, and Table 2) effectively constitutes a precision layer, which is necessary to implement because searching for relevant terms without applying fine-grained domain and concept specific knowledge would mostly return documents that are concept irrelevant. The step includes the application of rules such as combining multiple tagged concept-related words in close proximity to filter documents, identifying relevant windows of context, and paragraph/sentence starts/stops to remove obvious false positives. For example, when searching for the clinical concept of “allergy”, it is not enough to search for drug names, as these commonly occur in various contexts throughout the narrative. Concept relevant words may be subject to dependency structures for true positive detection (see Table 2, rule 4). In the case of drug allergies, while we do want to detect sentences like “The patient is allergic to Penicillin,” we are not so much interested in sentences such as “The patient is described Metformin for…”. Likewise, certain documents or paragraphs may be particularly concept relevant and demand careful attention to details when tagging and extracting text (Table 2, rule 1 and 6). For instance, by detecting the document types and the headlines occurring throughout the text, specific rules may be leveraged to analyze areas of the text with particular attention. When searching for e.g. the clinical concept “allergy”, a headline which includes the word “allergies” most of the time means that what follows may be relevant to examine meticulously for information about drug allergies by applying explicit concept relevant rules.

**Table 2:** A subset of the 35 rules used by the system to detect relevant allergy information

Rule	Description	Comments
1. Document filtering	Documents must contain concept-related words, or else they are filtered out [7, 32]	E.g., for the clinical concept of “allergy”, documents must contain concept-related words associated with e.g. “allergy”, “allergen” “allergic reaction” or “symptom”
2. Paragraph Boundary	Concept-related words must be located within the same paragraph [81, 82]	E.g., in case an “allergy”, “reaction” or “symptom” is identified in another sentence, a check for conformity with rule 2 and 3 is initiated again
3. Window of context	Allergy concept-related words must be located within the same sentence, or if located in adjacent sentences must be in proximity (within a ± 6 word distance), of other identified allergy concept-related words [9, 69]	Distance tolerance can easily be adjusted in the system. We experimented with different scopes. As also reported by Afzal et al. [5], we found a six to ten word distance to be optimal
4. Dependency	Concept-related words can be of type [32] 1) Exist alone 2) Primary (exist when supported by 1 or 3) 3) Secondary (depend on 2 for existence)	E.g., for the clinical concept of “allergy”: while words of type 1 (strong indicators like e.g. “Anaphylaxis”) are allowed to “exist alone” in a sentence, other types must conform to rules 2 and 3
5. Part of compound words	In case concept-related words are found as part of seldom-used compound words, they are also highlighted [69, 83, 84]	E.g., “Cave information”
6. Header detection	Specific rules apply for relevant text detected below headers until the start of the next detected paragraph [9, 69]	E.g., in case of “Allergies” header, all allergy concept-related words (with certain limitations) should be highlighted
7. Highlight color	The degree of word concept-relatedness determines (from low to high) text highlight color yellow, orange or red [81]	E.g., allergy concept-related words are highlighted in the text
8. Disambiguation	Often repeated words where non-conceptual meaning (“word sense disambiguation”) is alluded are filtered out [9, 18]	E.g., «the patient reacts to light» in eye examination reports
9. Negation	Detection of positive/negative contexts is handled by checking for the existence of negations in the text [6, 33]	E.g., “reacts to Penicillin” versus “does not react to Penicillin”
10. Permutations	Use of the word permutations dictionary may be enabled or disabled [13, 69]	An algorithm detects and stores the most used misspellings not already covered by the clinical knowledge base
11. Omitted documents	EHR documents of certain types or with certain headers or contents may be left out [28, 83]	Documents which, e.g., contain specific sensitive information may be left out of the results for some users
12. Concept search access control	Performing search for specific clinical concepts may be assigned or restricted to a group of users	Group of users may be defined in a flexible way E.g., a clinical department or individual users

To achieve granular contents control, and thus high-precision search results, the system embeds “If–Then” rules together with flexible regular expression (RegEx) based rules. An example of a simple “If–Then” rule is one that conditions the application of a set of specific deterministic rules according to which clinical concept is being searched for (see Algorithm 3).

**Figure:**
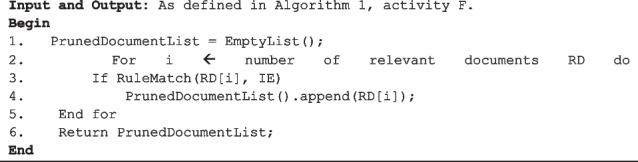
**Algorithm 3.** Function RuleBasedPruning(RD, IE).

A RegEx-based rule is a sequence of characters that defines search patterns (see Algorithm 4). Regex-based rules have been successfully applied in prior clinical NLP-related research, including e.g. NegEx and MedEx [6, 9].

**Figure:**
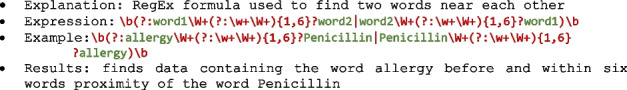
**Algorithm 4.** Proximity search algorithm.

**Fig. 9:**
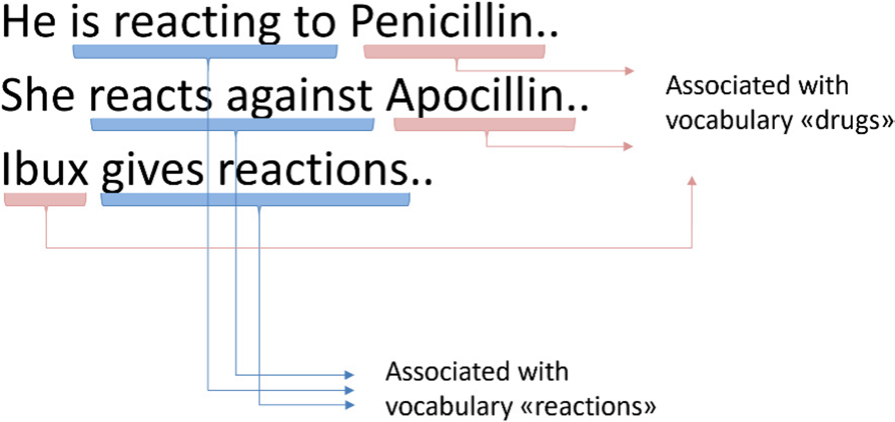
The dependency rule. Exemplified for the concept of “allergy.” Detection of “drugs” vocabulary words is dependent on detecting “reaction” vocabulary words in close proximity

The management module is used to define rules for detecting the location of concept-related words and their relations in the text. The defined rules are automatically/programmatically converted into RegEx-based search algorithms which are applied at run-time. Both the distance between concept-related words, whether they occur in the same sentence or in different sentences, their order and their dependence on each other are taken into consideration. Particularly, the so-called “Paragraph boundary” (rule 2), “Window of context” (rule 3), and the “Dependency” (rule 4) rules in Table 2 working together are central to achieving a precise search mechanism. For example, by applying the “Dependency” rule to a concept, it can be defined whether concept-related words have the power to stand-alone in the text (e.g., the word “anaphylaxis” when searching for the concept of “allergy”), or whether detection depends on another concept being detected in close proximity (see Fig. 9).

### Run-time utilization of the combined method for clinical concept searching and classification

Health professionals working for example in the out-patient’s clinic or in the operating theatre typically use the system to search for and classify clinical concepts in the patient EHR (step 6 in Fig. 1). The process starts with a health professional selecting a patient and a clinical concept by interacting with the system's GUI. Based on the selected clinical concept’s stored seed words of (i.e., stored at concept build time), the knowledge base is queried for relevant vocabulary and the EHR is retrieved. While the building of vocabulary mimics the process previously described for semi-supervised building of vocabulary (step 4 in Fig. 1), the clustering to group sets of coherent vectors in the knowledge base is now performed on-the-fly for all of the included vocabularies. For instance, the clinical concept of "Allergy" uses the seed words stored for its 23 dependent vocabularies to quickly generate a total defining vocabulary of about 7000 words. The system can optionally store a clinical concept’s total vocabulary “offline” for VSM independence or for export to other NLP tasks. However, run-time generation of vocabulary from scratch is preferable as it occurs almost instantaneously and ensures the automatic inclusion of new vocabulary after updated word embedding models have been implemented.

To perform clinical concept searching and classification, the system’s inference engine amalgamates the clinical concept relevant knowledge (i.e., stored in the knowledge base) with the relevant patient data stored in the EHR. Reflecting step 4 and 5 in the pipeline architecture (see Fig. 1), concept classification is essentially a two-step process. First, using the generated concept relevant vocabulary, a preliminary keyword-based searching and scoring of patient EHR data is performed. The documents in the selected patient’s narrative are scored according to the exact mixture of phrases from the clinical vocabulary they contain, probabilistically measuring the presence of the concept (see activity E in Algorithm 1, and Algorithm 5). All EHR data that does not contain concept relevant vocabulary is filtered out, and a much smaller sample is left for further in-depth rule-based concept analysis. Second, selected deterministic rules (see Table 2) are applied for fine-grained concept analysis. The partitioning of the process into two steps helps with transparency (i.e., the result of each process can be explained to the user) and also speeds up the classification process by ensuring we do not waste CPU-cycles on analyzing EHR data that does not contain concept relevant vocabulary. Finally, the concept relevant EHR data is presented to the user as a filtered list of documents, where concept relevant information is automatically focused (successively scrolled to) and highlighted for each of the viewed documents (see activity F in Algorithm 1, and rule 7 in Table 2, and Fig. 2).

**Figure:**
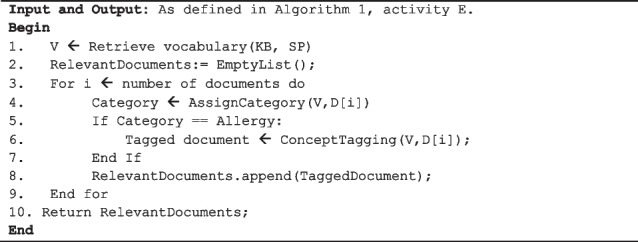
**Algorithm 5.** Function Categorize (KB, SP, D).

As noted, the combined method has been implemented in a CDSS (ICCS) we developed for clinical concept searching and classification. Using a dataset of authentic clinical notes, we empirically evaluated our combined method through several methods of analysis [1]. The results demonstrate that the method (see Figs. 1 and 2) is able to support clinical decisions by effectively filtering and summarizing information in allergy concept relevant clinical documents with a high degree of recall (92.6%) and precision (88.8%). The use of the system in clinical practice further allowed clinicians to identify patients at risk for allergy and provide early interventions that would prevent or mitigate the associated morbidity and mortality [68]. In the experiments we perform here (see Table 4), the combined method reaches state-of-the-art performance, with respectively 18.4% and 23.8% better precision and recall scores than the best performing pure machine learning-based method we evaluate.

### Experiments

We perform empirical experiments to evaluate the performance of the combined method on medical datasets containing complete real-life patient EHRs derived from Sørlandet Hospital Trust.

The combined method proposes to use unsupervised produced word embeddings to semi-automate the building of specialized clinical vocabulary. A condition for this is that methods employed for producing word embeddings are able to learn and represent the semantics of EHR-derived datasets. Complementing limited earlier research on the subject, we evaluate the quality of variously produced word embeddings on the task of word similarity by using clinically relevant (i.e., allergy related) seed terms as input.

Second, we evaluate the combined method on a clinically relevant classification task against other commonly used supervised learning methods. Based on the content of patient narratives, we perform classification of whether patients have allergies or not. This basically translates to the task of text classification, which typically sorts documents into a fixed number of predefined categories. The task here is much hardened, however, by the fact that classification has to be performed on the collective of each patient’s EHR documents. Each EHR contains large volumes of complex structured longitudinal data, including clinical events that are unevenly distributed over time [85]. To better understand the significance of our method’s rule-based component on performance, we also include extended results for the best scoring supervised method where unsupervised word embeddings are used as extra word features [50]).

### Dataset construction

The dataset we use for evaluation is non-public and derived from Sørlandet Hospital Trust’s enterprise EHR system. The dataset constitutes the clinical notes (863 937 documents, ∼1.2 billion tokens, and a dictionary of 863 937 unique unigram tokens or 1 803 428 common phrases) of 9267 patients that were admitted to the hospital for orthopedic surgical procedure performed between January 1^st^ 2014 and December 31^st^ 2015 (i.e., the study population). We employ the whole dataset to train the unsupervised algorithms on the task of word similarity, and a manually curated subset of the dataset (i.e., the subset dataset) to evaluate performance on the task of classification.

The subset dataset was produced by two health professionals (an anesthetist and a nurse with special training) who annotated randomly selected complete patient EHRs for allergy relevant information [86]. The annotations were registered, analyzed and systematized into categories of keywords reflecting that there had either been an “allergy” or an “allergic reaction” of some kind (allergy related terms and phrases, regular expressions consistent with allergic reactions, and symptoms confluent with allergy), together with ten categories constituting different types of reactive allergens. We next used this information to manually abstract and classify each of the patient EHRs into either an allergy “Yes” or “No” category. Thus, the labeled dataset effectively constitutes a binary classification problem. Next, the subset dataset was reduced in size to 430 randomly subsampled patient EHRs (76 319 documents, ∼15 951 K words, and ∼104 M tokens in total) to achieve a sufficiently balanced dataset. Finally, the dataset was prepared for compatibility with Weka (ARFF file format), Python (csv file format), and StarSpace (fastText file format), using C# to generate the necessary file formats.

### Experimental setup

All of the experiments were conducted on an Ubuntu 18.04 LTS × 64 server with Intel i7-8700 K 12-core CPU, 64 GB of memory, 2 TB SSD, and Nvidia GeForce GTX 1080 Ti GPU.

#### Word embedding experiments

We employed StarSpace, GloVe, Python Gensim (Word2vec and fastText CBOW and Skip-Gram variants), together with our own method using distributional semantics to train word embeddings on the whole dataset. To evaluate the quality of the learned word embeddings, we perform word similarity evaluation where the task is to measure how well word vector representations capture the notion of word similarity according to humans [63]. Using allergens as input words to each method, we rank the 10 and 200 nearest word vector pairs (k ∊ {10} ∧ (k ∊ {200}) according to their trained cosine similarities. We compare the produced word vector pairs against a manually curated taxonomy of allergens (the gold-standard). The gold-standard was developed by health professionals in the hospital [1, 87, 88], and contains approximately 7000 allergens classified as belonging to either drug, environmental, or food categories. There are currently no agreed-upon metric for evaluating word embeddings, and most suggested methods contain some element of qualitative analysis [54]. We used precision at K (Precision@K), corresponding to the percentage of relevant results in retrieved results, as metric to evaluate word similarity [54, 89]. I.e., if a word embedding method predicts n_p_ words and n_r_ words among them are relevant, the performance equals:

2 $$Precision@{n}_{p}=\frac{{n}_{r}}{{n}_{p}}$$

We experimented with different NLP pre-processing techniques (e.g., lowering case, punctuation removal and tokenization) and various hyperparameters such as dimensionality of word vectors, minimum word frequency, epochs, negative sampling (Word2vec and fastText), window size, and learning rate (alpha) to obtain optimal models. Word vectors require significant amounts of memory and storage [90]. Based on Pennington et al.’s observation that word vectors larger than 300 dimensions produce small returns [43], we restricted length to 300 dimensions. Stop words are not removed because they may render significant meaning to word vector representations. Assuming that they are close to optimal, we set any unspecified hyperparameters to their default values. The final set of hyperparameters used for the word embedding experiments are given in Table S1 in the supplemental material (Additional file 1: Supplemental Section 1). Finally, we also report the elapsed time for producing 100 dimensional word embeddings for all the methods.

We further implemented the best performing supervised CNN 1D neural network model with the best performing GloVe, StarSpace, Word2vec and fastText CBOW and Skip-Gram pre-trained word embedding models (using 100 and 300 word vector dimensions) to examine how such semi-supervised approaches perform on the classification task against other pure supervised methods and our own combined method [50].

#### Classification experiments

We used Weka v3.9, StarSpace [26], and Python code with implementations from the Scikit-learn, Keras, and Tensorflow libraries for the classification experiments. The classifiers we employed in Weka were Naïve Bayes, decision tree (J48/C4.5-based decision tree algorithm, Logistic regression (Multinomial logistic regression), Random forest, kNN (Instance-Based k—a nearest neighbors variant), SVM (Sequential Minimal Optimization—an SVM variant), and MLP (DL4jMlp). The classifiers we implemented in Python include close to standard vanilla long short-term memory (LSTM) [91], LSTM CNN, Bidirectional LSTM (Bi-LSTM), Bi-LSTM CNN, Bi-LSTM with Attention Mechanism [92], and Gated recurrent unit (GRU) [93]. For GRU and LSTM, we used the Nvidia CuDNN versions for increased GPU performance. Auto-Weka and random search were used in Weka for hyperparameter optimization, while Hyperas/Hyperopt random search and TensorBoard scalars and histograms were used in Python to identify optimal RNN/LSTM and CNN neural network specific parameter values such as filter size, hidden layers, dropout rate, back-propagation learning rate, and batch size. We also experimented with using different optimizers such as Adam, Adagrad, Adamax, Rmsprop, and Stochastic Gradient Descent (SGD), including parameters to control gradient clipping (clipnorm), momentum (SGD), and Nesterov momentum (SGD). The final results of the LSTM and GRU networks (except for the Bi-LSTM Attention variant) use Rmsprop with a learning rate of 10^–4^ [94], while most of the CNN networks adopts Adam with a learning rate of 10^–3^. However, we reduce the learning rate when the validation loss has stopped improving for 10 steps, as a strategy to prevent missing local minima during training in case of a loss plateau. For StarSpace, we employed a combination of random search parameters and default parameters. Again, unspecified hyperparameters were set to their default values based on the assumption that they are close to optimal. The final set of hyperparameters used for the classification experiments are given in Table S2 in the supplemental material (Additional file 1: Supplemental Section 2).

NLP-preprocessing in Weka, StarSpace, and Python (NLTK and Scikit-learn libraries) included cleaning and tokenization of the input data into unigrams and quadrigrams (e.g., the StringToWordVector filter in Weka). We adjusted relevant parameters (as closely as possible) to replicate the steps in Fig. 1 describing our combined method’s NLP pre-processing pipeline (including lowering case, removal of non-alphanumeric characters, sentence boundary detection and splitting, minimum term frequency 10) to ensure a fair comparison. For the conventional methods, we used the InfoGainAttributeEval method to determine the most significant attributes. For the neural networks (except MLP), we converted the EHRs into sequences and truncated or padded them respectively at a length of 39 000 (equaling the average length of the EHRs).

We refer to true positive (the number of items correctly predicted as belonging the positive class), false positive (the number of items incorrectly predicted as belonging to the positive class), true negative (the number of items correctly predicted as belonging the negative class), and false negative (the number of items incorrectly predicted as belonging to the negative class) as respectively TP, FP, TN, and FN. We further employ macro averaged precision, recall, and F-measure as evaluation metrics for the text classification task as follows:

3 $$Precision=\frac{TP}{TP+FP}$$

4 $$Recall =\frac{TP}{TP+FN}$$

5 $$F-measure= \frac{2 x Precision x Recall}{Precision+Recall}$$

We used StarSpace’s debug information to calculate a confusion matrix for each run, from which we derived macro averaged precision, recall, and F-measure scores. The results are expressed in terms of percentage (%), and the corresponding standard deviations are indicated after the “ ± “ symbol. The best obtained results are shown in boldface.

We experimented with different training, validation, and test dataset splits. All the conventional supervised methods and StarSpace were evaluated using tenfold cross-validation repeated 10 times with a 90% training / 10% test data split. For the LSTM, GRU, and CNN neural network variants, we implemented 100-fold cross validation, and in addition used a 10% validation dataset derived from the 90% train dataset split to provide unbiased evaluation of model fit on the training dataset, early stopping, and for tuning model hyperparameters. We monitored validation loss, and saved the best weights for each model. Training stopped after 150 epochs or once there was no improvement on the validation set for more than fifteen epochs. We calculated results for each fold based on the best working model (i.e., using the best stored weights). Finally, we report averaged scores over all the folds to estimate a predictive model for each of the models.

The classification results for our combined method were obtained by using ICCS to retrieve allergy concept relevant information for all of the patients in the subset dataset. Each of the 430 patient cases was manually classified into either an allergy “Yes” or “No” category based on system allergy status. Following this, evaluation of our combined method’s performance could be performed against the labeled subset dataset similarly to the other methods.

## Results

### Evaluating the quality of learned word embeddings

**Fig. 10:**
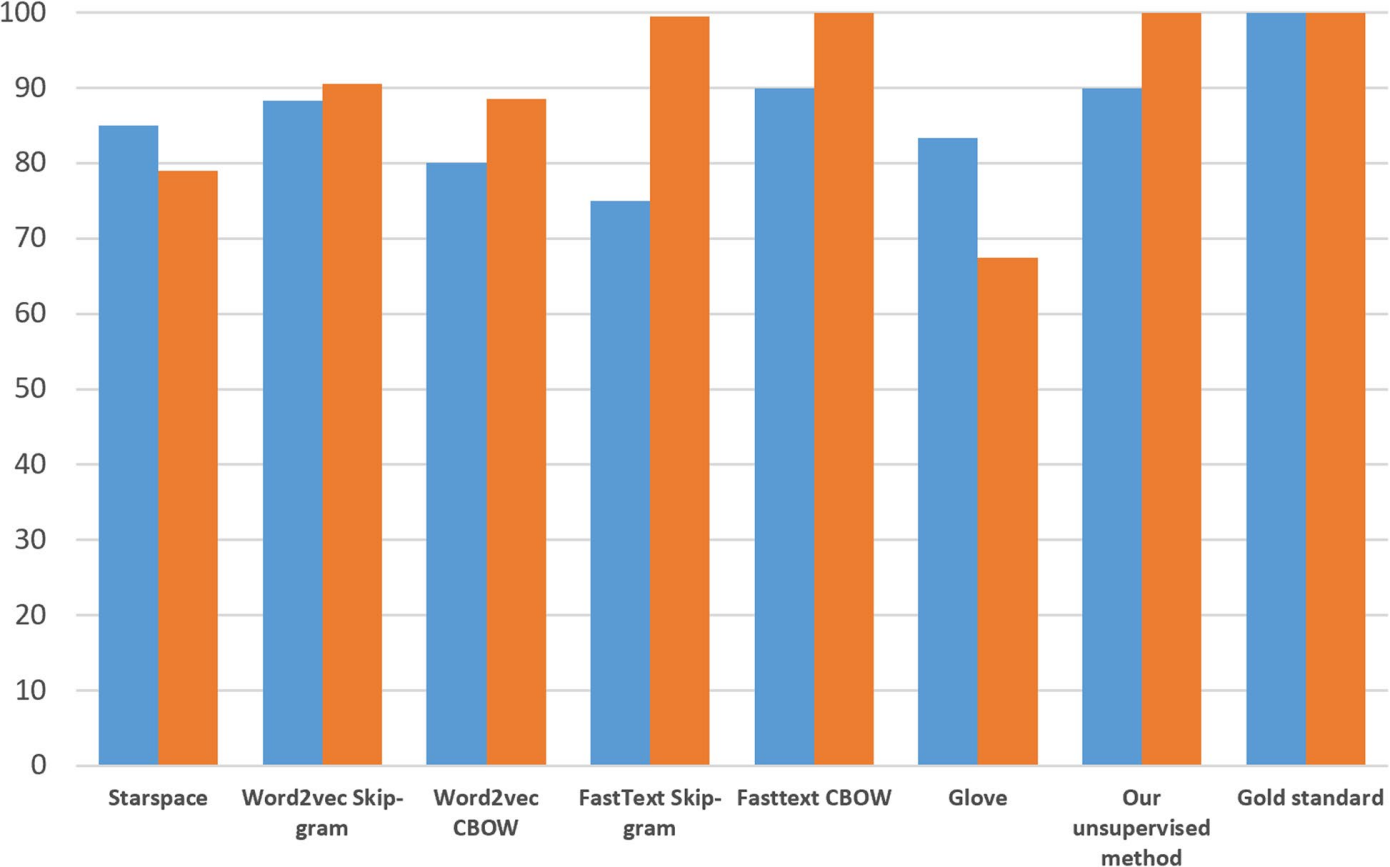
Normalized precision scores for word similarity according to the gold-standard. Blue series (Precision@30): 10 most similar words according to allergen categories drugs, environmental, and food. Orange series (Precision@200): extended 200 most similar words according to allergen category drugs

Employing the gold-standard containing manually classified allergens, we randomly picked the words “Apocillin”, “Cat”, and “Cultured milk” from each of the three categories of allergens (drugs, environmental, and food). Table 3 shows the results (precision@k, (k ∊ {10}) of the word embeddings models we evaluate, according to each of the allergen categories, where words (or part of phrases) that are co-occurring in the gold-standard are emphasized. We distinguish several measures of precision. Generally, only words that are fully matched in bold are counted as true positives. Phrases that are only partially matched are in bold italics and are given half score. In addition, to harden the precision task for the food allergen category, only foods that are drinkable liquids are given full score. Non-liquid items are marked in bold italics and are given half score. Similarly, only genuine environmental allergens are given full score in the environmental allergy category. Related allergens such as e.g. related to metals are marked in bold italics and are given half scores. The blue series in Fig. 10 reflects the total precision score (precision@k, (k ∊ {30}) in percent for each of the methods in Table 3. To also examine how precision holds up with more distant neighbors, we extend the experiment to the top 200 neighboring words for the frequently used drug “Apocillin” (precision@k, (k ∊ {200}). Only returned phrases that contain the name of a drug are counted as true positives (see the orange series in Fig. 10).

**Table 3:** Top 10 nearest neighbor (type of allergen-related) words returned by the unsupervised word embedding methods. Words co-occurring in the gold-standard similarity list of words are emphasized

	Type of allergen					
Method	Drugs: Apocillin		Environmental: Cat		Food: Cultured milk	
Our unsupervised method	**Apocillin** mg	**Paracet**	**Cat** and	**House dust**	**Biola**	**Orange juice**
	**Apocillin** tab	**Dalacin**	**Cat dog**	**Mite**	**Applejuice**	***Oats soup***
	**Penicillin**	**Ekvacillin**	And** cat**	**Wormwood**	**Apple juice**	**Whole milk**
	**Imacillin**	**Diclocil**	**Dog**	**Pollen**	**Buttermilk**	Or** juice**
	**Ciproxin**	**Keflex**	**Timothy**	**Horse**	**Coffee**	**Juice**
StarSpace	**Ciproxin**	**Abboticin**	**Dog**	**Grass**	**Drink**	***Strawberries***
	**Doxylin**	**Amoxicillin**	**Mite**	**Wormwood**	***Omelet***	**Biola**
	**Diclosil**	**Selexid**	**House dust mite**	**Dust**	***Scrambled eggs***	***Piece of bread***
	**Imacillin**	**Diclocil**	**Mold fungus**	**House dust**	***Youghurt***	***Cereal***
	**Apocillin**	**Erymax**	**Pollen**	Nuts	***Dessert***	***White bread***
Word2vec Skip-gram	**Doxylin**	**Abboticin**	**Dog**	**Timothy**	***Fishballs***	Drunk** juice**
	**Imacillin**	**Ciproxin**	**House dust Mite**	**Wormwood**	**Orange juice**	***Tomato***
	**Amoxicillin**	**Bactrim**	**Mold fungus**	**Dog cat**	**Buttermilk**	***Cereal***
	**Ery Max**	**Penicillin**	**Grass**	**Cat dog**	Extra** lowfat milk**	**Buttermilk**
	**Diclocil**	**Keflex**	**Pollen**	**Birch**	***Cocoa***	***Cucumber***
Word2vec CBOW	**Imacillin**	**Ery max**	**Dog**	**Mold fungus**	***Chocolate pudding***	Two cups
	**Ery Max**	**Penicillin**	**Wormwood**	**Dog cat**	**Juice**based	***Pancake***
	**Amoxicillin**	**Weifapenin**	**House dust mite**	**Animals**	***Cauliflower soup***	***Semulina porridge***
	**Doxylin**	**Keflex**	**Guinea pig**	**Cat horse**	***Yogurt***	***Cream of rice***
	**Abboticin**	**Diclocil**	**Cat dog**	**Grass**	***Crispbread***	Whole dinner
fastText Skip-gram	**Imacillin**	**Apocillin** 1 g	Treasure	Splashed	**Milk**	Gl **lowfat milk**
	**Amoxycillin**	**Bactrim Apocillin**	**Dog cat**	In at	**Milk juice**	**Soy milk**
	**Amoxicillin**	**Doktacillin**	**Mite cat**	Batt	**Orangejuice**	**Chocolate milk**
	**Acillin**	**Dicillin**	**Cat** bite	Tax arrears	**Orangejuice**	**Whole milk**
	**Doxycillin**	**Imacillin**	**Cat hair**	Padding	**Rice milk**	**Chocomilk**
fastText CBOW	**Imacillin**	**Apocillin**g	**Dog**	**Cat housedust**	**Whole milk**	**Chocomilk**
	**Apocillin**	**Bactrim Apocillin**	**Cat dog**	**Cat hair**	***Yogurt***	**Chocolate milk**
	**Apocillin Bactrim**	**Apocillin** 1 g	**Cats**	**Animals**	***Jogurt***	**Lowfat milk**
	**Ery max**	**Apocilin**	**Dogs cats**	**Wormwood**	***Chocolate pudding***	**Orange juice**
	**Amoxicillin**	**Doktacillin imacillin**	**House dust mite**	**Mite**	Chocolate taste	**Buttermilk**
GloVe	**Imacillin**	**Dalacin**	**Dog**	**Wormwood**	***Semolina porridge***	***Pommes frites***
	**Ciproxin**	**Bactrim**	**Mold fungus**	**Cat and dog**	***Cauliflower soup***	Drunk** juice**
	**Doxylin**	**Ery max**	***Nickel***	**Grass**	***Caramel cream***	***Gruel***
	**Penicillin**	**Keflex**	**Dog** and** cat**	**House dust mite**	**Coffee**	***Crispbread***
	**Diclocil**	**Selexid**	**House dust**	**Horse**	***Oatmeal***	**Buttered milk**

We observe that most of the 10 nearest neighbor n-grams returned by the different word embedding methods in Table 3 are co-occurring in the gold-standard. Thus, the returned terms are mostly analogous (i.e., synonymous) to the input allergen keywords. Word2vec CBOW and fastText Skip-Gram, return a few non-allergen terms such as e.g. “treasure,” “splashed,” “whole afternoon,” and “two cups.” As can be observed in Fig. 10 (see the blue series), fastText CBOW, Word2vec Skip-gram, and our own unsupervised method return the highest precision scores, followed by GloVe, Word2vec CBOW, StarSpace, and fastText Skip-gram. According to Mikolov et al. [39], an advantage with CBOW over Skip-Gram is slightly better accuracy for frequent words, especially when used on larger datasets. A probable factor favoring fastText CBOW on this complex dataset is that it is able to enrich word vectors with sub-word character n-grams. For example, the word vector "Penicillin" is here broken down into the separate word vectors "Peni" and "cillin". This capability benefits word embeddings in Fig. 10 (the orange series) for rare words (e.g., misspelled words such as “Penecillin”), or even related “cillin” ending drugs not seen during training. While fastText Skip-Gram also gains much by this capability, the added information from irrelevant char n-grams may actually be contributing to the method’s low precision@30 score. Supporting the latter, is that the sub-word character unit “att” is part of all of the non-allergen terms returned as synonymous environmental allergens in Norwegian. The inclusion of most of these non-allergen terms were found not to be related to specific hyperparameter settings, as they prevailed across different configurations. Scoring of the two other categories was not affected similarly, where fastText Skip-gram, on the contrary, was the most precise method. Specifically, this can be observed for the food allergen category, where it is the only method capable of only returning foods that are liquid.

We also observe the distinct dissymmetry of scores between the Word2vec and fastText Skip-Gram and CBOW variants in Fig. 10 (see the blue series). Recall that CBOW learns to predict a word by looking at the context of surrounding words, while Skip-Gram predicts the context from the input word. The result is somewhat surprising, as we had thought that dataset peculiarities would favor either a Skip-gram or CBOW approach irrespective of the chosen method. However, if we omit fastText Skip-gram’s misses of environmental allergens in Table 3, results would have been in-favor of Skip-gram for both methods.

Sahlgren and Lenci [95] showed that some matrix models can be superior to neural embeddings when trained on smaller datasets. We observe that GloVe on this dataset scores better than two of the prediction-based methods (see Table 3 and the blue series in Fig. 10). However, it fails quite badly when more distant neighbors are introduced (see Fig. 10, the orange series scores). While the fastText-based variants and our probabilistic approach are able to retain high precision scores for the 200 nearest neighbors, GloVe over the course introduces progressively more non-drug related words. This effect is still pronounced whether we use 100 or 300 word vector dimensions for GloVe. Although increasing the word vector dimension size generally helps with precision, a finding with all the methods is that longer distances increases the likelihood of identifying neighboring words that are non-drug related. Pennington et al. [43] were able to outperform prediction-based methods using GloVe on semantic tasks. However, they used the Word2vec CBOW and Skip-gram methods with only one iteration over the dataset, while using multiple iterations (as here) has later been shown to greatly improve performance [96]. The rather weak results for GloVe compared to the other prediction-based methods here corroborates with the earlier findings of Baroni et al. [34].

Combining the different measurements, the performance of StarSpace on this dataset is about average. We were somewhat surprised that StarSpace would not score closer to fastText on the extended word similarity task. StarSpace was created in a later iteration by the same research group that invented fastText (Facebook AI research), and Wu et al. found it to perform similar to fastText in their seed paper on StarSpace [42]. However, according to Facebook AI Research, the methods have different algorithms underneath which use different parameters, and StarSpace has not been optimized yet for training word level embeddings specifically [97].

**Fig. 11:**
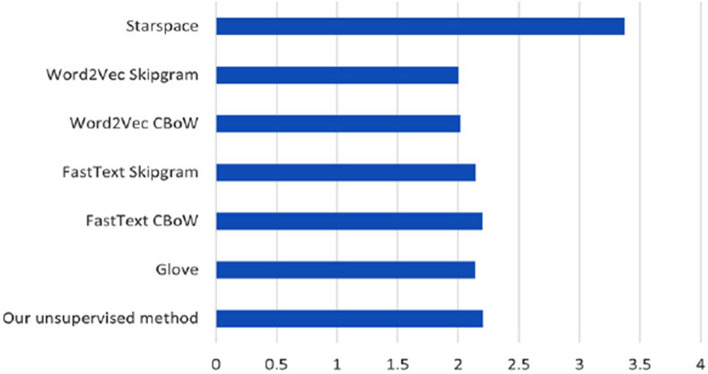
Runtimes in minutes (log scale) for the different unsupervised word embedding methods (using 100 dimensions)

Word embedding models used for clinical NLP must be regularly updated to keep the vocabulary relevant. Because the volume of clinical data available for training of such models in healthcare organizations typically may be much larger than what we use here, the total time it takes to train a word embedding model is important for practical feasibility. Figure 11 depicts the total elapsed training time for each of the word embedding models, while keeping key hyperparameters as similar as possible (i.e., using 100 word vector dimensions, a minimum word frequency of 10, 5 epochs, and window size of 5). Except for StarSpace that takes much longer to train, the small differences for the other methods are of little practical consequence. The time it takes to train the models are highly governed by specific hyperparameter settings. E.g., increasing the word vector size to 300 generally increased training time with a few hours for most of the methods, except for StarSpace where total elapsed training time increased to about 120 h.

The intention here is not on making conclusions as to what are better or worse methods for word embedding on medical datasets. While the results are far from comprehensive enough for such purposes, they also reflect that any such claim would probably depend on several variables. Performance gains of specific word embeddings may as observed in our experiment be due to certain system design choices (e.g., the inclusion of environmental allergies) and hyperparameter optimizations, rather than the choice of an embedding algorithm itself [98]. Furthermore, dataset domain specifics and size are known to influence results to a large degree, and the dataset we use here is not particularly large [51, 96]. However, we believe the experiments and the results are encouraging to the degree that they validate our proposal for using optimized word embedding models to support the construction of dictionaries of specialized clinical vocabulary for implementation in various downstream NLP tasks. Our empirical experiences with devising a CDSS implementing the proposed combined method to detect and classify patient allergies in EHRs show great usability at the level of precision achieved by fastText CBOW and our own probabilistic approach [68]. However, all of the methods benefit feature engineering to the extent that we believe they represent useful alternatives. The comparably higher training time for StarSpace, though, is a disadvantage and may limit practical applicability. Based on the findings, we suggest trying out different methods to determine which ones yield the best results in a particular context. As evidenced by earlier research [50], we also anticipate that even greater precision may be achieved by combining different word embeddings, as such furthering the case for cross-method usability.

### Evaluation on the task of classification

Table 4 displays the average classification results for the evaluated methods. All methods perform better than random, suggesting that they contain valid classification information.

**Table 4:** Average classification results for the evaluated methods. Results are presented in the format of mean percentage value ± SD at 95% confidence interval

Method	Precision	Recall	F-measure
Naïve Bayes	66.4 ± 1.7	70.5 ± 2.0	68.4 ± 1.6
Logistic regression	69.0 ± 1.8	59.6 ± 1.9	64.0 ± 1.5
Decision tree	64.7 ± 1.8	61.2 ± 2.4	62.9 ± 1.7
Random forest	62.8 ± 2.0	54.8 ± 2.0	58.5 ± 1.7
kNN	53.5 ± 3.2	29.1 ± 2.1	37.7 ± 2.3
SVM	66.7 ± 2.0	53.7 ± 2.0	59.5 ± 1.6
MLP	62.1 ± 2.1	61.9 ± 2.3	62.0 ± 1.6
StarSpace	62.4 ± 1.9	63.8 ± 2.2	63.5 ± 2.0
LSTM	63.9 ± 1.1	64.3 ± 1.3	64.1 ± 1.0
1D-CNN	69.3 ± 1.8	67.9 ± 1.6	68.6 ± 1.7
LSTM CNN	62.3 ± 1.9	62.7 ± 1.4	62.3 ± 1.7
Bi-LSTM	65.9 ± 1.5	65.2 ± 1.5	65.6 ± 1.5
Bi-LSTM CNN	64.1 ± 1.4	64.0 ± 1.4	64.1 ± 1.4
Bi-LSTM Attention	66.5 ± 2.1	67.2 ± 1.5	66.7 ± 1.9
GRU	65.5 ± 1.5	65.1 ± 1.5	65.3 ± 1.5
CNN 1D + fastText Skip-Gram 100 dims	60.7 ± 1.7	59.1 ± 1.3	59.9 ± 1.5
CNN 1D + fastText Skip-Gram 300 dims	63.3 ± 1.3	63.1 ± 1.4	63.2 ± 1.3
CNN 1D + fastText CBOW 100 dims	66.1 ± 1.3	63.9 ± 1.3	65.7 ± 1.2
CNN 1D + fastText CBOW 300 dims	74.2 ± 1.5	74.1 ± 1.5	**74.1 ± 1.5**
CNN 1D + Word2Vec Skip-Gram 100 dims	63.5 ± 1.9	61.5 ± 1.4	62.4 ± 1.7
CNN 1D + Word2Vec Skip-Gram 300 dims	70.7 ± 1.5	69.5 ± 1.4	70.1 ± 1.3
CNN 1D + Word2Vec CBOW 100 dims	61.7 ± 2.2	57.5 ± 1.0	59.2 ± 1.5
CNN 1D + Word2Vec CBOW 300 dims	66.1 ± 1.7	64.1 ± 1.3	64.5 ± 1.5
CNN 1D + Glove 100 dims	67.2 ± 1.3	67.2 ± 1.2	67.2 ± 1.2
CNN 1D + Glove 300 dims	71.3 ± 1.1	69.3 ± 1.3	70.3 ± 1.2
CNN 1D + StarSpace 100 dims	64.6 ± 1.5	63.0 ± 1.5	63.7 ± 1.4
CNN 1D + StarSpace 300 dims	70 ± 1.6	68.1 ± 1.6	68.9 ± 1.6
Our combined method	**92.6 ± 4.2**	**97.9 ± 2.4**	**95.1 ± 2.5**

As Fig. 4 illustrates, according to Zipf’s law [76], there are only very few irrelevant features in text categorization. This leads to the presumption that a good classifier should be able to combine many features, and that aggressive feature selection may result in a loss of information. Because we use a complex feature set (i.e., natural language), this should benefit neural networks that are able to cope with large feature spaces. As observed, plain 1D-CNN (68.6 ± 8.6) yielded the highest F-measure score of all the purely supervised methods. When also combined with pre-trained word embeddings, we were able to achieve even higher scores for 1D-CNN. The highest F-measure score (74.1 ± 1.5) was achieved by using the 300 dimensions fastText CBOW model, which also was the top performer in the word similarity task. Interestingly, a qualitative analysis reveals several more such scoring correlations. For example, Word2Vec Skip-gram also scores high on both tasks, while Word2vec CBOW and fastText Skip-gram scores relatively lower on both. Recall that in the case of fastText Skip-gram, the word embeddings for the environmental allergies category contained several out of place words. We take this as an indication that careful manual analysis of word embeddings may potentially also provide information of actual prediction performance. Another expected observation is that the increasing accuracy of pre-trained word embeddings that comes with using more dimensions seems to impact classification results positively. Given the small size (e.g., few examples, but high variance) and complex nature of the dataset (e.g., multiple misspellings), it is also likely that fastText CBOW scores extra on the classification task because of its ability to enrich word vectors with sub-word character n-grams [44]. Also note that not all of the 33 pre-trained word embedding models (all produced on our in-domain medical corpora, but with different hyperparameter settings) we experimented with benefitted recall and precision. We conclude that using pre-trained word embedding can help classification results on this dataset in some cases, but that results are highly sensitive to the methods and hyperparameters used for producing the actual word embeddings [98].

Another notable observation is that although the performance difference is not large, the Recurrent neural network (RNN) based methods we evaluated (LSTM and GRU variants) scored somewhat lower than the plain 1D-CNN variant. While the performance of GRU on modelling of many phenomena has been found to be similar to that of LSTM [93], GRUs have, however, as reflected by the results here (plain LSTM versus GRU) been shown to exhibit better performance on smaller datasets [99]. Combining LSTM with CNN, with the idea being that the CNN is used as a feature extractor for the LSTM, did not yield improvements over their plain LSTM counterparts on this dataset. However, contrary to Kim et al.’s [100] character-level implementation, which achieved results on par with existing state-of-the-art results on the English Penn Treebank dataset, our sequence implementation does not use a highway network and is only at word level. By adding an attention mechanism layer on top of the Bi-LSTM, we were able to increase the performance somewhat, but not enough to catch up with the score of the plain 1D-CNN. Furthermore, somewhat depending on exact model configurations, the plain 1D-CNN roughly completed learning five times faster than its plain LSTM (CuDNNLSTM) and GRU (CuDNNGRU) counterparts. We thus conclude that 1D-CNN is a strong performer on this particular dataset. This may be due to the fact that word order is of less importance than feature detection (e.g., searching for terms) when classifying this dataset, where CNNs have been shown to work particularly well for the latter [101].

Interestingly, among the conventional methods we evaluate, Naïve Bayes and Logistic regression score relatively high with F-measures of respectively 68.4 ± 1.6 and 64.0 ± 1.5. As most text classification problems are linearly solvable problems [102], it is reasonable to assume that these classifiers gain from their simple linear structure in this experiment. Unlike nonlinear models (e.g., neural networks), this may prevent fitting of training data too closely, which has been shown to benefit performance in many complex real-world situations [103].

Furthermore, the dataset we employ has a small sample size of only 430 EHRs (however, each EHR has a large set of documents). Because of its oversimplified assumptions, Naive Bayes generally requires a small amount of training data for model estimation [104], much less than its more complex neural network-based competitors [105]. Decision tree-based methods are used extensively in medicine due to their superior interpretability, which allow the immediate extraction of the decision process [106, 107]. However, the variants we implement here are only able to achieve F-measure scores of respectively 62.9 ± 1.7 (J48) and 58.3 ± 1.7 (Random Forest) on the dataset. The relatively weak recall and precision performances of the decision tree based algorithms are corroborated by previous research where they have been found to be outperformed in accuracy by other, less-interpretable machine learning models [106]. Decision tree-based algorithms such as the J48 algorithm generally implements a pruning technique to build a tree. While pruning may simplify the tree, and thus potentially benefit interpretation of results and help to generalize to new samples by removing overfitting data, it may also lead to poor accuracy in predictions. Note also that SVM, which Wang et al. [20] recently found to be the most frequently applied machine learning method in clinical information extraction applications, is not a particularly strong contender in this context. We also observe that kNN fails catastrophically on the dataset with a score of 37.7 ± 2.3. Generally speaking, however, the relatively good scores of several of the conventional methods (versus the more complex neural network based ones), coupled with their simplicity, interpretability, and small demands for technical infrastructure may explain why these have been and still are considered attractive choices in healthcare [20].

Finally, our combined method, using both unsupervised and supervised machine learning methods in combination with rules, outperforms all the other methods, with a precision score of 92.6 ± 4.2, recall score of 97.9 ± 2.4, and an F-measure score of 95.1 ± 2.5.

## Discussion

### An argument for using combined methods to obtain better predictions for clinical NLP

Both traditional rule-based and machine learning-based NLP systems have drawbacks that limit the feasibility of such systems in healthcare. Combined methods, such as the one we present here, may be perceived as a synthesis that endeavors to leverage the strengths of several methods while avoiding their weak spots. As previously described, ensemble and hybrid approaches for NLP have won machine learning competitions because they are able to produce results with high accuracy. Our combined method has documented high performance, and has also demonstrated its value in clinical practice [1, 68]. By combining multiple high-performing algorithms, we are able to obtain better predictive performance than could be obtained from any of the constituent algorithms alone. The combined method implements unsupervised and supervised machine learning for clinical concept building and pattern recognition, and deterministic rules for fine-grained control.

As demonstrated by the results, unsupervised word representations are potentially very useful in NLP tasks both as inputs to supervised learning algorithms and as extra word features in NLP [108], but current approaches suffer various shortcomings. Using a single point in a vector space to represent a word simplifies a concept or issue so that its nuance and complexity are lost, or important details are overlooked, limiting the usefulness for downstream NLP-tasks. While excelling at detecting complex patterns in text corpora, machine learning still lack the contextual and temporal awareness of humans. EHRs basically contains longitudinal, patient-based reporting of their clinical experience, and as such for successful classification purposes they must be analyzed in multiple dimensions (e.g., time and clinical context) and at multiple levels (e.g., document, paragraph, sentence, and word window). Although a clinical event such as patient allergy (the classification task here) described in the recent narrative of a patient may be of major importance for the correct classification of allergy status, it may be misleading in the case of another patient where it was mentioned 10 years ago but recently no longer applies. Modelling such temporal data has been shown to be extremely difficult for machine learning algorithms [85]. As observed in our own work, without the guidance of domain knowledge derived rules, learning algorithms may reject ambiguous domain relevant patterns as these in favor of “biased” but stronger patterns such as for example the name of a certain doctor who has treated many allergy patients. Detected patterns that cannot be related to domain knowledge are, however, usually of little relevance because they hold limited explanatory power for health professionals and patients. The use of domain knowledge derived rules on top of machine learning allows for fine-grained control of the detection of both contextual and domain relevant patterns, as it gives the ability to model complex relevant and dependent relationships. Rules makes it possible to operate at the EHR integral level, while simultaneously also take into consideration document, paragraph, sentence, and even word window levels of understanding to better model e.g. temporal events.

One can argue that given enough labeled data, nonlinear models (e.g., the neural networks used here) should be able to learn the fine-grained dependencies and patterns embodied by our method’s rules. However, large-sized labeled medical datasets (i.e., narrative focused) with the volume of samples needed to fine-tune data hungry neural networks for NLP are hard to come by. Due to medical domain experts being a scarce resource, the cost of obtaining enough labeled clinical data effectively renders the approach out of reach for most healthcare organizations. The process of creating the relatively small labeled dataset used in this work serves as an example in this respect. While the dataset only contains the EHRs of 430 patients (however, each EHR has a large set of documents), two health professionals still used about 3 months to analyze it for relevant allergy information. Furthermore, because of privacy issues, existing labeled medical datasets are mostly restricted to research purposes, and can seldom be shared across healthcare institutions. There are also interpretability issues with the results deep neural networks produce (i.e., “the black box”), and currently only a few healthcare institutions have the knowledge or technical infrastructure necessary for training such complex machine learning algorithms.

The combined method for clinical NLP that we have described also has other advantages, like potentially being language independent, automatically updatable (meaning minimum manual maintenance is necessary to build vocabularies and concepts), and able to cover misspellings, abbreviations, and acronyms. Finally, unlike a “black-box” type approach, by pushing raw data into an open processing pipeline that gradually refines the data, data transformations are made transparent and predictions interpretable. Input and output values from step to step may be monitored, traced, and judged, making the method highly suitable for implementation in for example CDSSs in healthcare [1, 68]. Such systems should ideally not only support clinical decisions; they should like human beings also be able to explain their decision making.

In conclusion, we believe the arguments put forth reflect the limits of pure machine learning-based approaches, including semi-supervised ones, for clinical NLP per se. In this respect, our combined method may be seen as a pragmatic attempt at using machine learning to augment traditional rule-based clinical NLP-systems that excel at accuracy and therefore are still mostly used. Our work leaves us with the insight that machine learning-based methods can play important roles in the context of clinical NLP, but that practical success to a large degree still depends on domain knowledge to derive precise vocabulary and rules.

### Limitations

A limitation of this research was that the EHR derived dataset we used has some weaknesses. First, the size of the dataset is only 1.2 billion tokens. Several of the neural network-based methods we evaluated would expectedly be favored by adding even more data. Pennington in their study for example, used both datasets containing 1, 1.6, 4.3, 6, and 42 billion tokens [43].

More data would also be a prerequisite for training large-scale Transformer-based (e.g., GPT and BERT) models [46, 47]. While we plan to further examine the effect on word embedding quality by training on larger medical datasets in the future, restrictions and regulations apply for accessing medical datasets that prevented us from doing so here [48]. Second, because the dataset is non-public, the results are not easily reproducible. Whereas benchmarking on public datasets is of interest to future research, this study is part of a larger research project in a Norwegian hospital where we have a particular focus on structuring the clinical (unstructured) Norwegian narrative by using automated methods. As described in the Background section, the medical language possesses characteristic features not reproduced in public datasets derived from other domains. As no readily available anonymized medical datasets exist in Norwegian, we had to construct our own dataset. The effective use of machine learning techniques depends on the characteristics of specific datasets [109]. Thus, an advantage is that our results have practical value and reliability for healthcare in the sense they are based on data derived from an empirical healthcare setting.

Our emphasis here was first on dissemination of the combined method, and not so much on achieving state-of-the-art results. As such, the experiments we performed were designed for simple setup and fair comparisons. Although we strived to achieve good performing hyperparameter configurations for all the included methods, it was impossible to fully traverse hyperparameter space for every method and variant. Therefore, unbeknown to us, better configurations may exist. However, the overall results are in-line with previous findings confirming the superior performance of combined methods (i.e., ensembles or hybrids) over other constituent algorithms alone [18].

A limitation with the combined method is that word embeddings trained in a particular healthcare organization setting cannot easily be transferred to other healthcare organizations. As discovered during our work, such models may contain sensitive information such as names, social security numbers, and phone numbers related to e.g. patients or health professionals. Currently, to our knowledge, no automated and reliable method for anonymizing VSMs exists. Therefore, besides the already described benefits of training word embeddings in the local context, for this reason it becomes imperative.

Finally, the combined method does not yet support linking of semi-supervised produced vocabulary with standardized medical terminologies such as SNOMED and ICD. We believe developing such mechanisms should be a prioritized next step with the system, as it would likely further complement the vocabulary building process and help further bridge the gap between structured data and unstructured information in EHRs.

## Conclusions

We have presented our novel combined method for clinical NLP, which implements unsupervised learning of word embeddings, semi-supervised learning for simplified and accelerated clinical vocabulary and concept building, and deterministic rules for fine-grained control of information extraction. The clinical language is automatically learnt with minimal manual feature engineering and tagging required from clinical experts to build vocabulary, concepts, and rules that supports a variety of clinical NLP downstream tasks. Effectively, the process renders the time-consuming manual annotation of the narrative obsolete. Employing a real-life EHR-derived dataset, we also evaluated various word embedding methods on the task of word similarity. We concluded that all of the produced models benefit feature engineering, and that they are capable of serving as the basis for further semi-supervised building of specialized clinical vocabularies that may be implemented for clinical NLP. The combined method’s performance was further evaluated on the task of classification against a range of common supervised learning algorithms, and achieved state-of-the-art performance. Unlike a “black-box” type approach, raw data is pushed into an open processing pipeline that gradually refines the data. Data transformations are thus made transparent and predictions interpretable, which is imperative for healthcare. The combined method also has other advantages, like potentially being language independent, demanding few domain resources for maintenance, and able to cover misspellings, abbreviations, and acronyms. Based on the promising results we have achieved by combining unsupervised, supervised, and rule-based algorithms, we suggest more research should be aimed at exploiting the inherent synergies between these paradigms for clinical NLP. Plans for implementing the combined method in more advanced CDSS to aggregate information for diagnosis purposes in the hospital are now being discussed.

### Supplementary Information

**Additional file 1.**

## Data Availability

There are ethical restrictions on sharing the study’s data (the data contains potentially sensitive information). In accordance with restrictions imposed by the Regional Committees for Medical and Health Research in Norway (approval no. 2016/329), data must be stored on a secure server at Sørlandet Hospital Trust. The contents of the ethics committee's approval resolution as well as the wording of participants' written consent do not render open public data access possible. Access to the study's data may be requested by contacting Geir Thore Berge (first author) at Sørlandet Hospital Trust (postmottak@sshf.no).

## Abbreviations

ANN: Artificial neural network
BERT: Bidirectional encoder representations from transformers
Bi-LSTM: Bidirectional long short-term memory
CBOW: Continuous Bag-of-Words model
CDSS: Clinical decision support system
CRF: Conditional random field
EHR: Electronic Health Record
FN: False negative
FP: False positive
GloVe: Global Vectors for Word Representation
GPT: Generative Pre-training
GRU: Gated recurrent unit
GUI: Graphical user interface
ICCS: Information System for Clinical Concept Searching
ICD: International Classification of Disease
kNN: k-nearest neighbor
LSTM: Long short-term memory
NLP: Natural language processing
RegEx: Regular expression
RNN: Recurrent neural network
SGD: Stochastic Gradient Descent
SNOMED-CT: Systematized Nomenclature of Medicine Clinical Terms
SVM: Support Vector Machine
TN: True negative
TP: True positive
VSM: Vector space model
