# Supplementary material for: Combining unsupervised, supervised and rule-based learning: the case of detecting patient allergies in electronic health records
Source: BMC Med Inform Decis Mak. 2023 Sep 18;23:188. doi: 10.1186/s12911-023-02271-8 (PMC10507898; doi:10.1186/s12911-023-02271-8)
Supplement: Supplementary file 1 — Additional file 1. [file 12911_2023_2271_MOESM1_ESM.docx]

# Supplemental Section 1: Table S1

The table S1 shows the final set of hyperparameters used to produce the results for each of the evaluated algorithms in Table 2.

**Table S1**

The final set of hyperparameters used for the results shown in Table 2.

| Algorithm | Hyperparameters |
| --- | --- |
| All | All the algorithms make use of NLP-preprocessing techniques such as: removal of non-alphanumeric characters; replacement of capitals with small letters; removal of punctuations/sentence boundary detection and splitting; tokenization; minimum term frequency of 10; automatically detect common phrases/word n-grams. |
| Word2vec CBOW | size=300, window=6, iter=15, min_count=10, workers=12, sg=1,  alpha=0.025, max_vocab_size=None, sample=0.001, seed=1, min_alpha=0.0001, hs=0, negative=5, ns_exponent=0.75, cbow_mean=1, sorted_vocab=1, batch_words=10000, compute_loss=False |
| Word2vec Skip-Gram | size=300, window=5, iter=15, min_count=10, workers=12, sg=0, alpha=0.025, max_vocab_size=None, sample=0.001, seed=1, min_alpha=0.0001, hs=0, negative=5, ns_exponent=0.75, sorted_vocab=1, batch_words=10000, compute_loss=False |
| fastText COW | size=300, window=5, iter=15, min_count=10, workers=12, sg=1, alpha=0.025, max_vocab_size=None, word_ngrams=1, sample=0.001, seed=1, min_alpha=0.0001, negative=5, ns_exponent=0.75, cbow_mean=1, min_n=3, max_n=6, sorted_vocab=1 |
| fastest Skip-Gram | size=300, window=5, iter=15, min_count=10, workers=4, sg=0,  alpha=0.025, max_vocab_size=None, word_ngrams=1, sample=0.001, seed=1, min_alpha=0.0001, negative=5, ns_exponent=0.75, min_n=3, max_n=6, sorted_vocab=1 |
| GloVe | vector_size=300, learning_rate=0.05, window-size=15, epochs=30, no_threads=12,  vocab_min_count=10 |
| StarSpace | -normalizeText 0 -minCount 10 -ngrams 4 –epoch5 -trainMode 5  -dim 300 -lr 0.01 -negSearchLimit 50 -maxNegSamples 50 -loss hinge -margin 0.05  -similarity cosine -adagrad 1 -ws 5 -batchSize 5 -thread 12 |
| Our own word embedding method | window=10, ngrams 4 |
|  |  |

# Supplemental Section 2: Table S2

The table S2 shows the final set of hyperparameters used to produce the results for each of the evaluated algorithms in Table 4.

**Table S2**

The final set of hyperparameters used for the results shown in Table 4.

| Algorithm | Hyperparameters |
| --- | --- |
| All  All CNN, LSTM, and GRU variants. | All the algorithms make use of NLP-preprocessing techniques such as: removal of non-alphanumeric characters; replacement of capitals with small letters; tokenization; minimum term frequency of 10.  ReduceLROnPlateau(monitor='val_loss', factor=0.5, patience=10)  EarlyStopping(monitor='val_loss', patience=15, mode='auto') |
| Naïve Bayes | weka.classifiers.bayes.NaiveBayes (default) |
| Logistic regression | weka.classifiers.ScikitLearnerClassifier -batch 100 -learner LogisticRegressionCV –parameters Cs=10, class_weight=None, cv=None, dual=False,fit_intercept=True, intercept_scaling=1.0, max_iter=100,multi_class='ovr', n_jobs=10, penalty='l2', refit=True, scoring=None, solver='lbfgs', tol=0.0001, verbose=0 |
| C4.5/J48 (Decision tree) | weka.classifiers.trees.J48 -C 0.1 -M 8 |
| Random forest | weka.classifiers.trees.RandomForest -P 100 -I 1000 -num-slots 12 -K 0 -M 1.0 -V 0.001 -S 1 |
| IBk (kNN) | weka.classifiers.lazy.IBk -K 4 -W 0 –A (unigrams) weka.classifiers.lazy.IBk -K 2 -W 0 –A (quadrigrams) |
| SMO (SVM) | weka.classifiers.functions.SMO -C 1.0 -L 0.001 -P 1.0E-12 -N 0 -V -1 -W 1 -K "weka.classifiers.functions.supportVector.PolyKernel -E 1.0 -C 250007" -calibrator "weka.classifiers.functions.Logistic -R 1.0E-8 -M -1 -num-decimal-places 4" |
| MLP (Multi-Layer Perceptron) | weka.classifiers.functions.Dl4jMlpClassifier -S 1 -early-stopping "weka.dl4j.earlystopping.EarlyStopping -maxEpochsNoImprovement 5 -valPercentage 10.0" -normalization "Standardize training data" -iterator "weka.dl4j.iterators.instance.DefaultInstanceIterator -bs 1" -iteration-listener "weka.dl4j.listener.EpochListener -n 5" -layer "weka.dl4j.layers.OutputLayer -activation \"weka.dl4j.activations.ActivationSoftmax \" -dropout 0.0 -name \"Output layer\" -lossFn \"weka.dl4j.lossfunctions.LossMCXENT \"" -config "weka.dl4j.NeuralNetConfiguration -biasInit 0.0 -biasLearningRate NaN -l1 NaN -l2 NaN -leakyreluAlpha 0.01 -learningRate 0.1 -learningRatePolicy None -lrPolicyDecayRate NaN -lrPolicyPower NaN -lrPolicySteps NaN -minimize -algorithm STOCHASTIC_GRADIENT_DESCENT -updater \"weka.dl4j.updater.Adam -beta1MeanDecay 0.9 -beta2VarDecay 0.999 -epsilon 1.0E-8\" -weightInit XAVIER" -numEpochs 70 -queueSize 0 -zooModel "weka.dl4j.zoo.CustomNet " |
| StarSpace | -normalizeText 0 -minCount 10 -ngrams 4 –epoch 100 –debug 1 –verbose true  –epoch5 -trainMode 0 -lr 0.01 -negSearchLimit 50 -maxNegSamples 50 -loss hinge -margin 0.05 -similarity cosine -adagrad 1 -ws 5 -batchSize 5 -thread 12 |
| LSTM | lstm size=32, return_sequences=True, dropout after (LSTM layer)=0.5, batch_size=16, epochs=150  RMSprop (lr=0.0001, rho=0.9, epsilon=None, decay=0.0) |
| 1D-CNN | filters=64, kernel_size=5, activation='relu', strides=1, dropout before (CNN layer)=0.2, dropout after (CNN layer)=0.2, hidden_dims=64, batch_size=32, epochs=150  Adam(lr=0.001, beta_1=0.9, beta_2=0.999, epsilon=None, decay=0.0, amsgrad=False) |
| LSTM CNN | filters=128, kernel_size=5, activation='relu', strides=1, lstm size=128, return_sequences=True, dropout before (CNN layer) 0.3746350041674067, dropout after (LSTM layer) = 0.583831565328610, batch_size=16, epochs=150  RMSprop (lr=0.0001, rho=0.9, epsilon=None, decay=0.0) |
| Bi-LSTM | lstm size=128, return_sequences=True, dropout before (LSTM layers)=0.3746350041674067, dropout after (LSTM layers)= 0.583831565328610, batch_size=16, epochs=150  RMSprop (lr=0.0001, rho=0.9, epsilon=None, decay=0.0) |
| Bi-LSTM CNN | filters=64, kernel_size =3, activation='relu', strides=1, lstm size=128, return_sequences=True, dropout after (LSTM layer)=0.5, batch_size=16, epochs=150  RMSprop (lr=0.0001, rho=0.9, epsilon=None, decay=0.0) |
| Bi-LSTM Attention | lstm size=128, dropout after (attention layer)=0.5, batch_size=64, epochs=150, return_sequences=True  Adam(lr=0.001, beta_1=0.9, beta_2=0.999, epsilon=None, decay=0.0, amsgrad=False) |
| GRU | GRU size=32, return_sequences=True, dropout after (GRU layer)=0.5, batch_size=16, epochs=150  RMSprop (lr=0.0001, rho=0.9, epsilon=None, decay=0.0) |
| CNN 1D+fastText Skip-Gram | filters=128, kernel_size=6, activation='relu', strides=1, dropout before (CNN layer)=0.4298755674328703, dropout after (CNN layer)=0.046848850006786014, hidden_dims=64, embedding_dims=100/300, batch_size=16, epochs=150  Adam(lr=0.001, beta_1=0.9, beta_2=0.999, epsilon=None, decay=0.0, amsgrad=False) |
| CNN 1D+fastText CBOW | filters=512, kernel_size=5, activation='relu', strides=1, dropout before (CNN layer)=0.2, dropout after (CNN layer)=0.2, hidden_dims=512, embedding_dims=100/300, batch_size=16, epochs=150  Adam(lr=0.001, beta_1=0.9, beta_2=0.999, epsilon=None, decay=0.0, amsgrad=False) |
| CNN 1D+Word2Vec Skip-Gram | filters=64, kernel_size=7, activation='relu', strides=1, dropout before (CNN layer)=0.2, dropout after (CNN layer)=0.2, hidden_dims=600, embedding_dims=100/300, batch_size=16, epochs=150  Adam(lr=0.001, beta_1=0.9, beta_2=0.999, epsilon=None, decay=0.0, amsgrad=False) |
| CNN 1D+Word2Vec CBOW | filters=512, kernel_size=5, activation='relu', strides=1, dropout before (CNN layer)= 0.6108763092812357, dropout after (CNN layer)=0.21280043312755825, hidden_dims=512, embedding_dims=100/300, batch_size=16, epochs=150  Adam(lr=0.001, beta_1=0.9, beta_2=0.999, epsilon=None, decay=0.0, amsgrad=False) |
| CNN 1D+Glove | filters=384, kernel_size=5, activation='relu', strides=1, dropout before (CNN layer)= 0.6108763092812357, dropout after (CNN layer)=0.21280043312755825, hidden_dims=384, embedding_dims=100/300, batch_size=16, epochs=150  Adam(lr=0.001, beta_1=0.9, beta_2=0.999, epsilon=None, decay=0.0, amsgrad=False) |
| CNN 1D+StarSpace | filters=512, kernel_size=5, activation='relu', strides=1, dropout before (CNN layer)= 0.1602501347478713, dropout after (CNN layer)=0.5350807190884803, hidden_dims=512, embedding_dims=100/300, batch_size=8, epochs=150  Adam(lr=0.0001, beta_1=0.9, beta_2=0.999, epsilon=None, decay=0.0, amsgrad=False) |
